# Diversity, Antimicrobial Action and Structure-Activity Relationship of Buffalo Cathelicidins

**DOI:** 10.1371/journal.pone.0144741

**Published:** 2015-12-16

**Authors:** Biswajit Brahma, Mahesh Chandra Patra, Satyanagalakshmi Karri, Meenu Chopra, Purusottam Mishra, Bidhan Chandra De, Sushil Kumar, Sourav Mahanty, Kiran Thakur, Krishna Mohan Poluri, Tirtha Kumar Datta, Sachinandan De

**Affiliations:** 1 Animal Genomics Lab, Animal Biotechnology Center, National Dairy Research Institute, Karnal, 132001, Haryana, India; 2 Department of Biotechnology, Indian Institute of Technology, Roorkee, Uttarakhand, India; Bose Institute, INDIA

## Abstract

Cathelicidins are an ancient class of antimicrobial peptides (AMPs) with broad spectrum bactericidal activities. In this study, we investigated the diversity and biological activity of cathelicidins of buffalo, a species known for its disease resistance. A series of new homologs of cathelicidin4 (*CATHL4*), which were structurally diverse in their antimicrobial domain, was identified in buffalo. AMPs of newly identified buffalo CATHL4s (buCATHL4s) displayed potent antimicrobial activity against selected Gram positive (G+) and Gram negative (G-) bacteria. These peptides were prompt to disrupt the membrane integrity of bacteria and induced specific changes such as blebing, budding, and pore like structure formation on bacterial membrane. The peptides assumed different secondary structure conformations in aqueous and membrane-mimicking environments. Simulation studies suggested that the amphipathic design of buCATHL4 was crucial for water permeation following membrane disruption. A great diversity, broad-spectrum antimicrobial action, and ability to induce an inflammatory response indicated the pleiotropic role of cathelicidins in innate immunity of buffalo. This study suggests short buffalo cathelicidin peptides with potent bactericidal properties and low cytotoxicity have potential translational applications for the development of novel antibiotics and antimicrobial peptidomimetics.

## Introduction

Almost all eukaryotes including fungi produce antimicrobial peptides (AMPs) as primary defence against bacterial pathogen [1, 2]. The diversity of antimicrobial peptides discovered is great; nonetheless, they commonly have low molecular weight (2–5 kDa), amphipathic design and positively charged membrane-acting characteristics [1].The cathelilcidin family represents an important and widely studied group of mammalian AMPs [3]. The family was named after their conserved N-terminal domain that shares high homology with cathelin, an inhibitor of the cysteine proteinase cathepsin L. On the contrary, the C-terminal antimicrobial domain (AMD) is highly variable and represents functional diversity of this family. Cathelicidins are initially translated as precursor pre-pro-peptides and are released as active AMPs following proteolytic cleavage. The mature peptides are unusually rich in certain amino acids such as proline, arginine, and tryptophan and exhibit a variety of secondary structures, *i*.*e*., α-helix, β-hairpin, and random coils [4]. These amphipathic peptides bind to the bacterial membrane and kill bacteria by different mechanisms that include formation of water-filled toroidal pore (*e*.*g*. LL-37) [5], alteration of cytoplasmic membrane, septum formation, and inhibition of DNA or protein synthesis (*e*.*g*. indolicidin) [6].

The diversity of cathelicidin AMDs is a consequence of gene duplication followed by subsequent divergence that had taken place in Laurasiatheria (hoofed mammals) and Xenarthra (e.g. Platypus, Tammer wallby) [4, 7]. In contrast, Euarchontoglires like human and mice have only a single cathelicidin gene encoding an α-helical cationic peptide [4]. This diversity also reflects pathogen-driven natural adaptation of different species to find their ecological niche. Water buffaloes (*Bubalus bubalis*) are acclimatized to thrive in harsh tropical and subtropical climates, wet grasslands, marshy and swampy areas of Indian sub-continent, Mediterranean regions, Caribbean, Africa, and South America. Along-with the excellent milk producing ability, buffaloes are well-known for their exceptional disease resistance [8, 9]. These animals are less susceptible to tick-borne diseases [8] and severities of diseases such as trypanosomiasis, tuberculosis, brucellosis, rinderpest and piroplasmosis are less deleterious in buffalo as compared to cattle [10]. Considering a high microbial burden in the natural habitat of buffalo, we hypothesized that cathelicidin gene family could be diverse in this species. Here, we studied the cathelicidin genes from buffalo population and identified novel cathelicidin AMPs. The antimicrobial potency of newly identified cathelicidin AMPs was evaluated and the mechanism by which the peptides exert their bactericidal action was deciphered.

## Materials and Methods

### Ethics Statement

Buffalo tissues were collected from the Municipal Slaughter House, New Delhi, India with permission for research use. The slaughter house follows all the ethical and humane standards for animal slaughter and is regulated by norms of Government of India. Electrical stunning followed by *Halal* slaughter is practiced for euthanasia of animals in this slaughter house. National Dairy Research Institute (NDRI), a government organization, is permitted to collect animal tissues for research use. Blood samples were collected from healthy female buffaloes maintained under standard management at the experimental animal herd of NDRI. Permission was taken from Institutional Animal Ethics Committee (IAEC) of NDRI. The samples were collected by skilled technicians after proper restraining of animals under the supervision of a veterinary officer present at the cattle yard, NDRI.

### PCR Amplification, Cloning and Single Strand Conformation Polymorphism (SSCP) Analysis

Primers for cathelicidin (*CATHL*) genes were designed from buffalo EST sequence information available in UCSC genome browser (Table A in S1 File). Two PCRs (25 μl each) were set up for each sample with a negative control. The reactions contained 1×Phusion HF Buffer (Thermo Fisher, PA, USA), 200 μM of each dNTP, 0.5 μM of each primer, and 1U Phusion DNA Polymerase (Thermo Fisher, PA, USA) and 100 ng genomic DNA as template. A touchdown protocol (annealing: 68–64°C, Δt -1°C/cycle, 5 cycle followed by 63°C for 25 cycle) was followed to amplify the *CATHL* genes. PCR products were purified with Charge Switch PCR purification kit (Invitrogen, CA, USA). Purified PCR products were cloned onto pTZ57R/T vector (Thermo Scientific, PA, USA). For all *CATHL* genes, both strands of at least five plasmids representing animals of different breeds were sequenced.

For SSCP analysis of *CATHL4* gene, purified full length *CATHL4* amplicons from 25 buffaloes representing five breeds: Murrah, Mehsana, Niliravi, Nagpuri and Bhadawari, were cloned onto pTZ57R/T vector (Thermo Scientific, PA, USA). At least 50 random clones for each amplicon were selected and plasmids were isolated. A short fragment (~170 bp) representing *CATHL4* exonIV was amplified from these plasmids. PCR products were denatured (95°C for 10 mins), snap cooled, and electrophoresis was carried out in non-denaturing polyacrylamide gel [12% A:B (37.5:1); 110V, 4h]. The gel was stained by silver staining protocol [11]. Representative plasmids showing different SSCP patterns were sequenced for full length *CATHL4* nucleotide sequence.

### Determination of Absolute Copy Number

The absolute copy number of buffalo *CATHL4* (*buCATHL4*) was determined by qRT-PCR, as previously described [12, 13]. For copy number estimation, a region near 3′ UTR of *CATHL4* was amplified and analyzed. Region spanning Exon I–Intron III was excluded because it shared a much conserved homology with other *CATHL* genes. The 12 bp indel region of CATHL4 Exon IV was also excluded as this could affect accurate quantitation of fluorescence. Briefly, concentrations of plasmids containing *CATHL4* inserts were adjusted at 10 ng/μL using NanoDrop 1000 Spectrophotometer (Thermo Fisher Scientific, Wilmington, DE, USA). A tenfold dilution series of the plasmid constructs were used to construct the corresponding standard curve. The concentration of the plasmid was converted to corresponding copy concentration using the following equation [14]

$$DNA\;copy=\frac{6.023\times 10^{23}\;(copies/mol)\times DNA\;amount(g)}{\left(Plasmid+Insert)length(bp)\times 660(g/mol/bp\right)}$$

The standard curve was constructed by linear regression of the plotted points of the logarithm of initial template copy concentrations against the crossing point (Cp) values of serially diluted plasmids. From the slope of each curve, PCR amplification efficiency (E) was calculated according to the following equation [15]:

$$E=10^{-1/slope}-1$$

To determine the absolute copy number of CATHL4 gene in genomic DNA samples of different breeds of buffalo, concentrations of all gDNA samples were adjusted to 10 ng/μL. All real-time PCR runs were performed in triplicate and the ‘crossing point’ or Cp values were determined by ‘second-derivative max method’ on a LightCyclerW 480 instrument with software version 1.5 (Roche Diagnostics, Mannheim, Germany). For normalization of data, standard curve was made from plasmids containing cloned fragment of β-casein (*CSN2*). *CSN2* was amplified simultaneously with *CATHL4* gene in genomic DNA samples. Normalization was done by obtaining a ratio of CATHL4 copy number: *CSN2* copy number.

### Antimicrobial Assays

The buCATHL4 peptides were synthesized by G L Biochem (Shanghai, China). Purity and molecular mass of the peptides were provided by the vendors using HPLC and mass spectrometry, respectively. The purity of all peptides were *>*98%. Minimum inhibitory concentrations (MIC) of buCATHL4s against the pathogenic microorganisms were tested by broth micro-dilution technique [16]. Briefly, bacteria were grown in Mueller Hinton broth (Sigma-Aldrich, MO, USA) at 37°C. The OD of early-log phase cultures were adjusted to 0.08–0.1 and further diluted (1:200) to keep inoculums concentration nearly 10^6^ colony forming units (CFU)/ml. Diluted peptides (10 μl) were mixed with equi-volume solution A (0.02% acetic acid containing 0.4% BSA) and were serially diluted in the solution B (0.01% acetic acid containing 0.2% BSA). Bacterial inoculums (90 μl) were mixed with serially diluted peptides and incubated at 37°C for 24 h without shaking. The lowest peptide concentration in the well that showed no visible turbidity was defined as MIC. All peptides were tested in triplicate. For time kill kinetics experiments, peptides (1×, 2× and 4× MIC) were added to bacterial suspension of *Staphylococcus aureus*, *Salmonella enterica* and *Pseudomonas aeruginosa*, as described in the MIC determination method. Aliquots of representative wells were diluted and plated on Muller Hinton agar plates at 0, 1, 4, 12 and 24 h intervals. Water with 0.01% acetic acid and ampicillin (100 mg/ml) were taken as positive and negative controls, respectively. The results were represented as log CFU/ml.

### Biofilm Assay

The efficacy of peptides against biofilms of *S*. *aureus* and *P*. *aeruginosa* was determined using previously described method [17]. Overnight grown bacterial cultures were diluted (1:100) and 200 μl suspensions were mixed with each peptide (1× and 2× MIC) in a 96 well plate and incubated for 24 h at 37°C. Biofilms were fixed with paraformadehyde solution, cultures were decanted and 0.1% crystal violet solution was added to stain the film. The plate was rinsed thrice with water and air-dried. The stain was dissolved by 100 μl of acetic acid (30%) and absorbance was measured at 570 nm. The experiment was performed in triplicate, 30% acetic acid was taken as a blank.

### Cell Membrane Permeability Assay

The damage to bacterial membrane following addition of peptide was assessed by BacLight kit (Life Technologies, CA, USA) [18]. Aliquots of *S*. *aureus*, *S*. *enterica* and *P*. *aeruginosa* cultures from exponential (shaking incubation for 2, 3, and 4 h, respectively) and stationary phases (overnight incubation) were harvested by centrifugation (5000 g for 10 min) and diluted in PBS. Peptides (200μM) were added to bacterial suspension and 50 μl aliquots were removed immediately or after 1 h incubation. Equi-volume mixtures of SYTO9/PI (3 μl/ml bacterial suspension) were added to these aliquots and fluorescent micrographs of stained bacteria were taken at 1000×magnification in a microscope (Olympus BX51 fitted with DP71 camera) with a fluorescence illuminator system.

### Scanning and Transmission Electron Microscopy

Early log phase cultures of *S*. *aureus* and *S*. *enterica* were subjected to different concentration of peptides and incubated at 37°C. The bacterial pellets after centrifugation (5000 g for 10 min) were washed with 0.1M cacodylate buffer (pH 7.2) and were fixed in 2.5% gluteraldehyde at 4°C for 2 h. For scanning electron microscopy, bacteria were dehydrated using gradient series of ethanol wash (30, 50, 70, 90 and absolute). The samples were air-dried overnight, gold coated (Eiko IB-3 ion coater), and viewed in a Zeiss *EVO* 18 *Special Edition* SEM. For transmission electron microscopy, fixed samples were adhered to hexagonal PELCO® Grids (Ted Pella, Inc. USA), washed once with Mili-Q water and stained with uranyl acetate (2%) buffer. Samples were viewed in a JEM 1011 (Jeol, Japan) microscope.

### Circular Dichroism (CD) Spectroscopy

CD spectroscopy was carried out with a Jasco J-715 CD spectrophotometer using a 1 mm path length. Spectra were collected between 190 and 250 nm wavelengths at 25°C at 1 nm intervals. Peptides were analyzed at 100 μg/ml concentration in 10 mM sodium phosphate (pH 7) and 20 mM sodium dodecyl sulfate (SDS) in 10 mM sodium phosphate (pH 7). Data of three scans per sample were averaged and expressed as [θ] deg cm^2^ dmol^-1^, which was calculated as follows: *Mean residue ellipticity* = [(*θ*_*obs*_/10) × (*MRW*/*c* × 1)], where θ_abs_ is the observed ellipticity at a given wavelength [mdeg], MRW is residue molecular weight (Mw/number of amino acids), c is peptide concentration (mg/ml), and l is the path length (cm).

### Molecular Dynamics (MD) Simulations

MD simulations were performed to study the mechanism of membrane deformation in the order of 100 ns time scales. As a representative peptide, buCATHL4B was simulated with model phospholipid membranes consisting of either the zwitterionic 1,2-dipalmitoyl-sn-glycero-3-phosphocholine (DPPC) or a more negatively charged 3:1 mixture of 1-palmitoyl-2-oleoylphosphatidylcholine (POPC) and 1-palmitoyl-2-oleoyl-sn-glycero-3-phosphoglycerol (POPG) lipids. Varying numbers (2 −8) of monomeric buCATHL4Bs peptides were initially placed asymmetrically in the aqueous phase close to one of the leaflets of the bilayer. All MD simulations were performed using the GROMACS 4.5 software package [19]. The Berger-lipid force field was used for phospholipids and Gromis96-53a6 parameters were used to represent the peptide interactions. Trajectory data analysis and structure visualization were performed using VMD 1.9.1 [20] and accessory plug-ins. The detail method of MD simulation can be found in Text A in S1 File).

### Measurement of Cytokine Response

For measurement of *in vitro* cytokine response, PBMCs were isolated from blood and maintained in serum-free RPMI-1640 (Sigma Aldrich, MO, USA) medium. Cells were exposed to peptides (500 nM), muramyl-di-peptide (Invivogen, San Diego, CA) [MDP (10 μg/ml) as positive control] and PBS (negative control). Cells were incubated for 1 h and relative mRNA abundance of cytokines was quantified by qRT-PCR using RPS-18 gene as housekeeping control. Total RNA, extracted by TRIzol (Ambion, CA, USA) method was used for cDNA preparation (Superscript III cDNA synthesis kit; Invitrogen, CA, USA).qRT-PCR reactions were performed in a Light Cycler® 480 II machine (Roche Diagnostics, USA). Each reaction consisted of 2 μl cDNA template, 5 μl of 2× SYBR® Green PCR Master Mix (Thermo Scientific, PA, USA), 0.25 μl each of forward and reverse primers (10 pmol) and nuclease free water for a final volume of 10 μl. Each sample was run in duplicate.

### Cytotoxicity Assay

The hemolytic activities of peptides were determined against buffalo red blood cells (RBC). Blood sample was collected in EDTA containing vacutainer. The buffy coat and serum fraction was removed after centrifugation at 300×g. RBC suspension was washed thrice with PBS buffer, and diluted to 1:10 ratio. Serially diluted peptides were added to 10% RBC suspension in a microtitre plate and incubated for 1 h at 37°C with gentle shaking. The plate was centrifuged at 1000×g for 5 min and the extent of haemolysis was determined by measuring the absorbance of supernatants at 540 nm. Triton X 100 (0.1%) and PBS were taken as complete (100%) and no lysis (0%) controls, respectively. Percentage of hemolysis was calculated using the following formula:

$$Hemolysis\;(\%)=\frac{{OD}_{576nm}\;of\;treated\;sample-{OD}_{576nm}\;of\;negative\;control}{{OD}_{576nm}\;of\;positive\;control-{OD}_{576nm}\;of\;negative\;control}\times 100$$

To assess toxicity towards fibroblast cells, buffalo primary foetal fibroblast cell culture was established as described previously by us [21]. Before addition of peptides, cells were trypsinized, washed twice in PBS and resuspended in PBS containing propidium iodide (PI, 2 μl/ml). Cell suspension (10^6^ cells/ml) was then added to serially diluted peptides in a microtitre plate. The change in membrane permeability of fibroblast cells was assessed by flow cytometry (BD FACSCantoII Cell Analyzer, BD Biosciences) by calculating proportions of PI positive cells. The experiment was carried out in duplicates.

## Results

### Buffalo Harbours Rich Repertoire of CATHL4 AMPs

**Fig 1:**
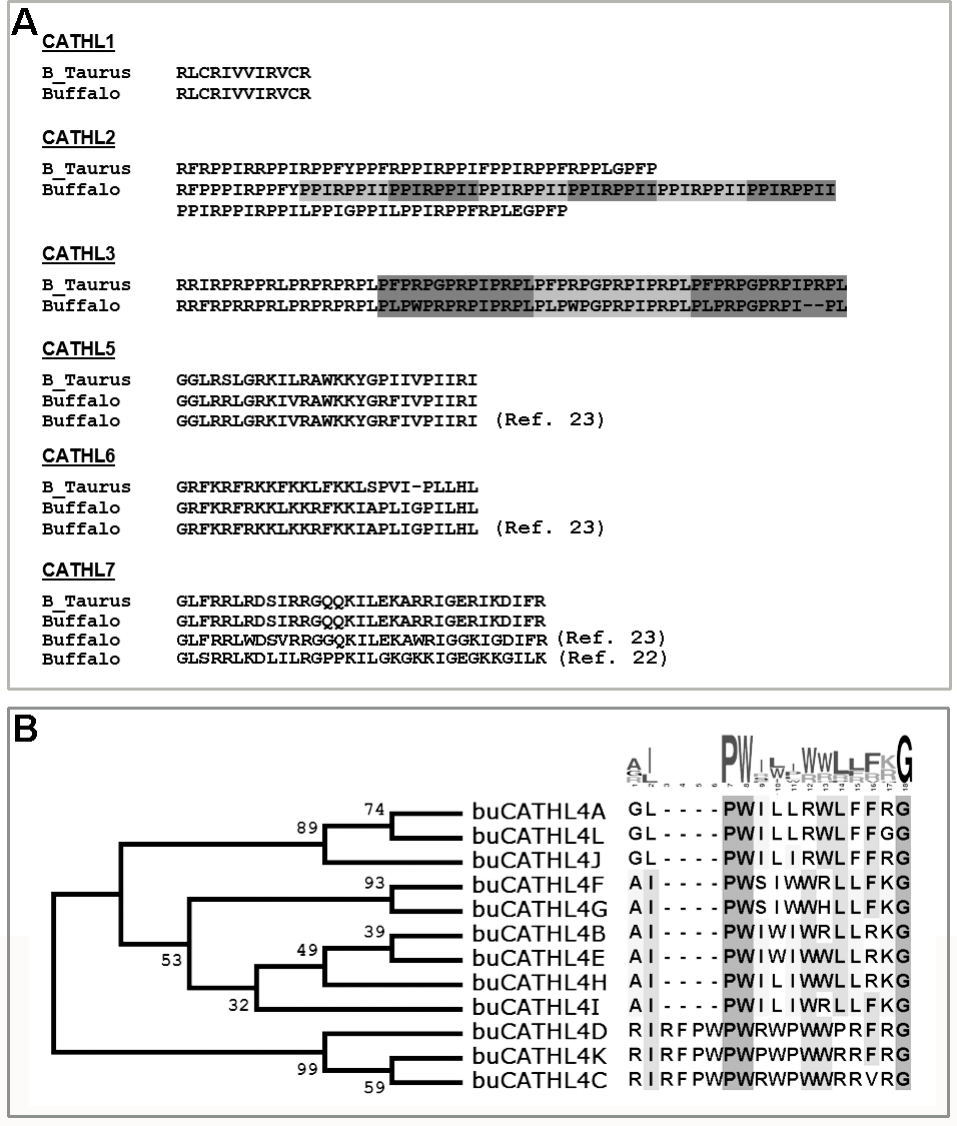
Repertoire of cathelicidin AMPs in buffalo. (a) Comparison of AAMP region of cattle and buffalo cathelicidins. (b) Phylogenetic relationship and consensus of amino acid residues of the 12 CATHL4 AMPs. The Neighbor-joining tree construction was based on complete nucleotide sequences of the CATHL4 variants (NCBI Accession ID: KJ173930—KJ173976).

The genomic organization of the *CATHL* locus varies across the species and often represents multiple copies of the gene with subtle or no difference in the AMD region (Fig A in S1 File). Using standard PCR amplification, cloning and sequencing strategy, seven *CATHL* genes (*CATHL1-7)*, each encoding a different AMP, were identified in buffaloes (NCBI Accession ID: KJ173977- KJ173982). Comparative analysis of cattle (*Bos taurus*) and buffalo *CATHL* sequences showed highly conserved homology in the exon I—intron III regions. Deduced amino acid sequences showed CATHL1 AMP was identical in cattle and buffalo (Fig 1A). Contrary to earlier reports [22, 23] that showed a significant difference in amino acid sequence between cattle and buffalo CATHL7 AMPs, our study showed buCATHL7 as close ortholog of cattle CATHL7. However, we observed a few major differences in the AMP region of other buffalo cathelicidins. For example, the buffalo CATHL2 AMP region was comparatively longer (94 aa) than the corresponding region in cattle (43 aa) and comprised of a repetitive motif (PPIIPPI/R). Buffalo CATHL3 AMP, like the cattle sequence contained a repetitive motif, but the motif had a few amino acid substitutions and/or deletions when compared to cattle sequence. Amino acid sequence of CATHL5 and CATHL6 AMPs found in this study, were identical to that had been reported earlier [23], but showed a few amino acid substitutions and an insertion, when compared to cattle orthologs.

Among seven buffalo cathelicidin genes studied, the most interesting was *CATHL4*, as the gene not only showed high polymorphism, but also indicated a possible copy number variation in buffalo. As preliminary sequencing results indicated a varying AMD region of buffalo *CATHL4*, therefore, the polymorphic status of the gene was screened by SSCP. Purified full length *CATHL4* amplicons from 40 buffaloes representing five breeds (Murrah, Mehsana, Niliravi, Nagpuri and Bhadawari) were cloned. At least 50 random clones for each amplicon were selected and plasmids were isolated. Thereafter, a short fragment (~170 bp) representing *CATHL4* exonIV was amplified from these plasmids and subjected to SSCP analysis. Corresponding plasmids representing different SSCP patterns (Fig B in S1 File) were sequenced for full length *CATHL4* gene. SSCP screening coupled with Sanger sequencing confirmed that *CATHL4* AMD region was indeed highly polymorphic in buffalo (NCBI Accession ID: KJ173930—KJ173976). Confining our analysis to the region that encodes mature CATHL4 AMP, a total of 12 subtypes of *CATHL4* were identified in the animals studied (Fig 1B). According to the frequency of occurrence of variants in studied population, these AMPs were designated as buCATHL4A (wild type) to buCATHL4L. Amino acid sequence of wild type buCATHL4A has been reported earlier [24], however, our study showed *CATHL4* could have several variants in buffalo. These buCATHL4 AMPs represented two major groups of structural variants, which differed in length by four amino acids.

It is understandable that two allelic variations are possible for a locus and PCR-SSCP coupled with cloning could show maximum three SSCP patterns, even for an animal carrying heterozygous alleles at more than one locus (Fig C in S1 File). However, to our surprise, SSCP analysis of multiple clones (~50 clones) from *CATHL4* amplicon of single animal showed more than three patterns (Fig D in S1 File). This findings were particularly evident with animals from Mehsana and Murrah breeds. Subsequent sequencing of clones showing different SSCP patterns revealed an individual animal could harbour 4–6 variants of *CATHL4*. Presence of multiple *CATHL4* variants in single animal indicated this was not simple allelic variation but the gene could be present in multiple copies (Fig C in S1 File). This finding was also supported by absolute quantitation of the *CATHL4* that showed that the gene had a high copy number in different breeds of buffalo (Table 1; Fig E in S1 File). A previous study has reported high copy number of *CATHL4* in indicine cattle [25], but its polymorphic status has not been explored. Taken together, our results are suggestive of a possible expansion and further divergence of *CATHL4* paralogs in buffalo. This being the case, repertoire of cathelicidin AMPs could be considered very rich in buffalo, compared to other domestic animals (Fig A in S1 File). We hypothesized that a pathogen-driven selection for innate resistance played its role in expansion of buffalo *CATHL4*, endowing superior antimicrobial spectrum and potency to the newly identified buCATHL4s over the prevalent wild type (CATHL4A). To test this hypothesis, we investigated biological properties of newly identified buCATHL4s.

**Table 1:** Copy number variation (CNV) of *CATHL4* gene in different breeds of buffalo.

Breed	No. of samples	log Copy No. (Mean ±SEM)	Copy No. (Mean ± SEM)	Normalized Copy No. (Mean ± SEM)
Bhadawari	04	6.420 ± 0.091	2.79E+06 ± 4.92E+05	5.117 ± 0.901
Mehsana	14	6.662 ± 0.005	4.65E+06 ± 5.51E+04	8.525 ± 0.101
Murrah	16	6.621 ± 0.010	4.41E+06 ± 9.07E+04	8.082 ± 0.166
Nagpuri	10	6.528 ± 0.008	3.44E+06 ± 6.84E+04	6.300 ± 0.125
Nili-Ravi	10	6.564 ± 0.006	3.69E+06 ± 4.90E+04	6.767 ± 0.089

### buCATHL4 AMPs Possess Broad Spectrum Antimicrobial Activity

Among the 12 newly identified CATHL4 AMPs, seven peptides (present in at least two out of five buffalo breeds studied) were selected for evaluation of their antimicrobial activity. Cattle CATHL4 (indolicidin) was used as control. MIC determination by broth micro dilution procedure revealed growth inhibition properties of buCATHL4s against selected G+ and G− pathogens (Table 2). The emerging problematic G+ pathogen like *S*. *aureus* [26, 27] and *L*. *monocytogenes* were susceptible to buCATHL4 peptides at low concentrations. buCATHL4B, 4C, 4D, and 4E showed lower MIC towards *E*. *coli* and also were very effective against *P*. *aeruginosa*, a G- bacteria often shows resistance to amphipathic drugs because of their outer membrane barrier [28]. Another G- pathogen *S*. *typhimurium* was also sensitive to most of the buCATHL4s at low concentrations.

**Table 2:** Minimum inhibitory concentration (MIC) of selected buffalo cathelicidin4 AMPs assessed by broth microdilution method.

Organisms	buCATHL4A	buCATHL4B	buCATHL4C	buCATHL4D	buCATHL4E	buCATHL4F	buCATHL4G	Indolicidin
	MIC (μM)							
*Stahylococcus aureus* ATCC*-29213	3	0.4	0.2	6	6	6	0.8	3
*Stahylococcus aureus* NCDC^#^-0111	25	0.4	0.4	12.5	0.4	6	12.5	1.5
*Listeria monocytogenes* ATCC-19111	6	12.5	1.5	6	6	3	1.5	6
*Bacillus cereus* NCDC-0240	100	12.5	0.8	1.5	0.8	100	50	0.8
*Pseudomonas aeruginosa* ATCC-27853	>200	0.4	0.2	25	6	100	200	25
*Pseudomonas aeruginosa* NCDC-0105	>200	3	6	50	6	50	200	12.5
*Salmonella typhimurium* ATCC-14028	100	6	3	50	1.5	0.2	3	12.5
*Escherichia coli* MTCC^§^-0723	200	6	12.5	6	6	>200	50	0.8
*Acinetobacter johnsonii* NCDC-0072	200	12.5	6	50	1.5	200	25	6

* ATCC: American Type Culture Collection

^#^NCDC: National Collection of Dairy Cultures

^**§**^MTCC: Microbial Type Culture Collection

**Fig 2:**
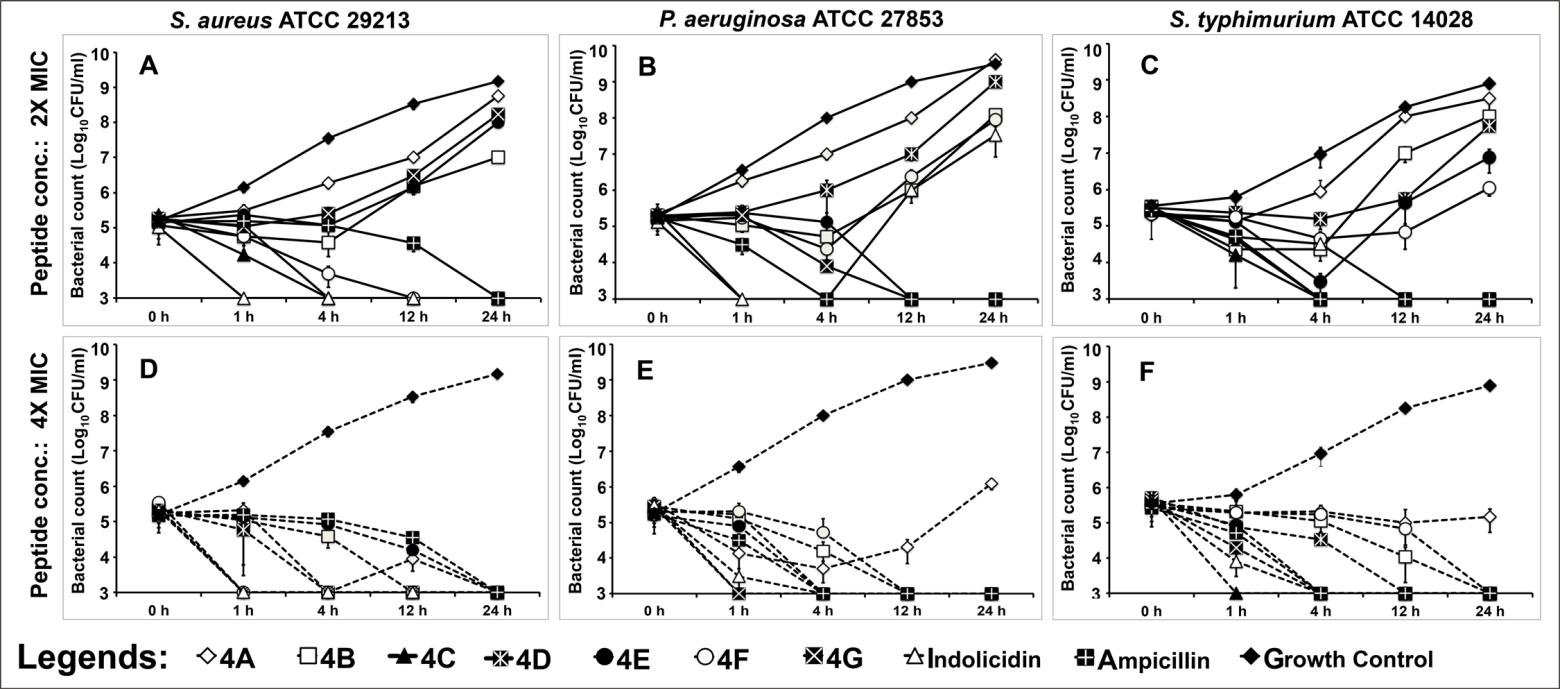
Time kill kinetics of buCATHL4 peptides against (A) *S*. *aureus* (B) *S*. *typhimurium* and (C) *P*. *aeruginosa* following addition of peptides at 2× and 4× MIC concentration.

The time kill kinetics assay revealed a dose- and time-dependent killing profile of buCATHL4s against three pathogens *viz*. *S*. *aureus*, *S*. *typhimurium* and *P*. *aeruginosa*. At 2×MIC, buCATHL4s showed major reduction in viable number of bacteria, but not all peptides showed complete elimination of test pathogens (Fig 2A–2C). For example, complete growth inhibition of *S*. *aureus* was observed only with buCATHL4C, 4D and 4F. However, at 4× MIC, a time dependent bactericidal effect was observed (Fig 2D–2F). buCATHL4C, 4D, 4E, and 4G showed a comparatively better killing rate than indolicidin and ampicillin. The G- *P*. *aeruginosa* was eliminated relatively earlier, but buCATHL4C, 4F, and 4G were able to eliminate *S*. *aureus* completely as early as 4 h.

**Fig 3:**
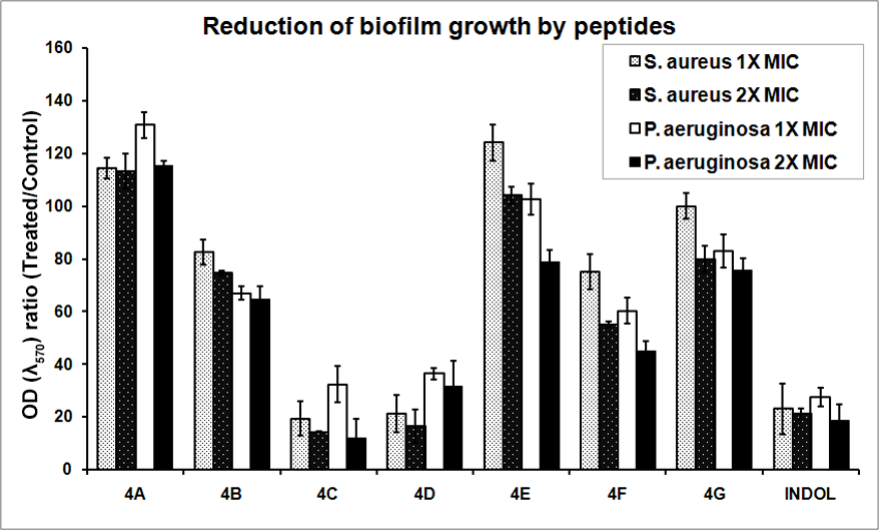
Reduction level of *S*. *aureus* and *P*. *aeruginosa* biofilm growth by peptides at 1× and 2× MIC.

The effectiveness of peptides was also evaluated against the biofilms of *S*. *aureus*, *P*. *aeruginosa*. The biophysical properties of the biofilm confer extreme antibiotic resistance to microorganisms [29]. Reduction in biofilm growth of both *S*. *aureus* and *P*. *aeruginosa* were substantial with buCATHL4C and 4D (Fig 3). At 2× MIC, buCATHL4F also reduced the biofilm growth of *P*. *aeruginosa* considerably. Indolicidin was also effective against both *S*. *aureus* and *P*. *aeruginosa* biofilms. Taken together, it was evident that antimicrobial potency and killing kinetics were different among buCATHL4s and selected peptides reduced growth of *S*. *aureus* and *P*. *aeruginosa* biofilms.

### Antimicrobial Action of buCATHL4s Is Mediated by Perturbation of Bacterial Membrane

**Fig 4:**
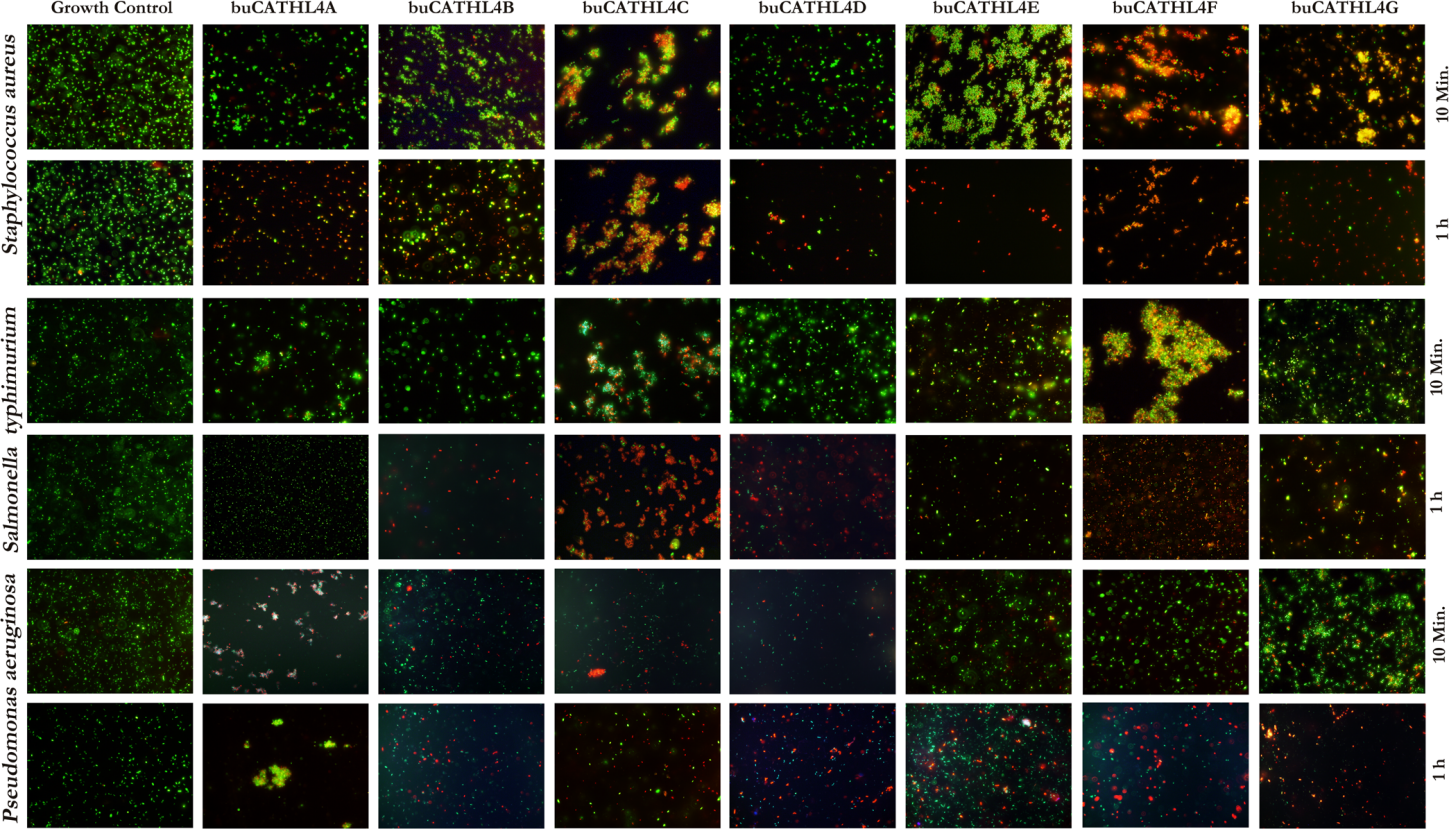
Peptide induced changes in membrane permeability of bacteria as assessed by SYTO9/PI dyes. Green fluorescence indicates live bacteria with intact plasma membrane. Red and yellow fluorescence indicates bacteria with altered permeability that had uptaken propidium iodide dye.

In order to assess the alteration of the membrane permeability of bacteria, LIVE/DEAD *Bac*Light viability assay (Molecular Probes, Oregon, USA) was performed after 10 min and 1 h of peptide addition. Peptide mediated disruption of bacterial membrane integrity was quick in some cases. A notable observation with this assay was immediate clumping of both *S*. *aureus* and *S*. *typhimurium* after addition buCATHL4C, 4E, 4F and 4G (Fig 4). Agglutination has been reported to be crucial for the antimicrobial function of eosinophilic cationic proteins [30] and amyloids [31]. Intrinsic aggregation properties of peptides promote agglutination of the bacteria through rearrangement of the proteins that expose the hydrophobic patch of peptide leading to disruption of the lipophilic barrier of the cells [31]. Consistent with this finding, a considerable proportion of cells within the clump showed altered membrane permeability (red and yellow) immediately after peptide addition. After 1 h, the clumps disintegrated along-with a reduction of viable cell number. Agglomeration was not seen for *P*. *aeruginosa*, but a fair proportion of cells were found to have altered membrane permeability after peptide addition. Peptide mediated changes in the bacterial membrane permeability were also evident with stationary phase cultures. As seen with log phase cultures, bacterial clumping was also observed with stationary phase cultures of *S*. *aureus* and *S*. *typhimurium*. The peptides had changed membrane permeability of stationary cultures of *S*. *aureus* and *P*. *aeruginosa* (Fig F in S1 File). buCATHL4B, 4C, 4D, and 4F also affected membrane permeability of latent stages of *S*. *typhimurium*, nevertheless, the proportion of cell affected were comparatively less. This indicated effectiveness of peptides against stationary phase cultures, which often possess drug-resistance properties and have been associated with chronic infection [32].

**Fig 5:**
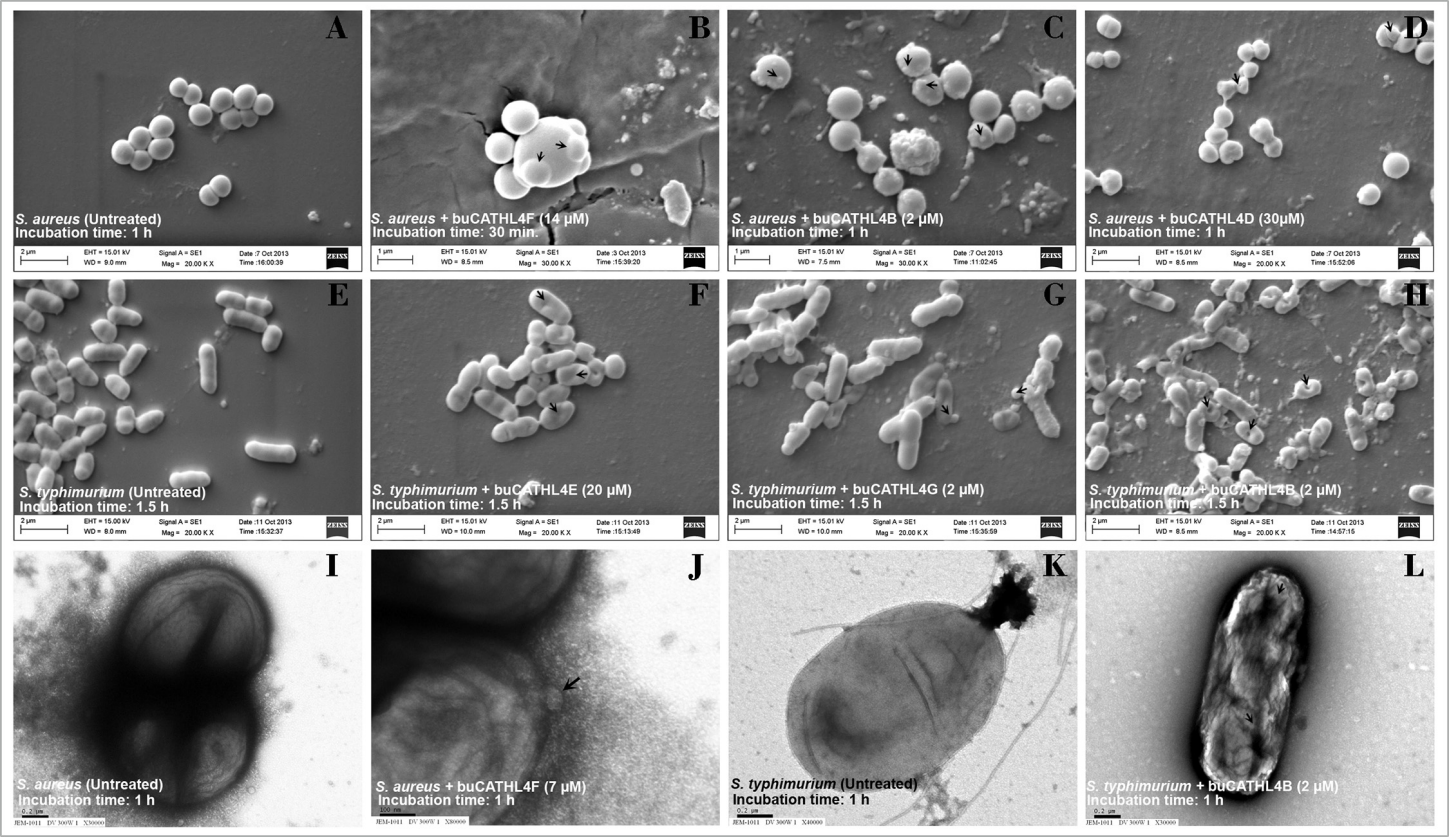
Scanning and transmission electron micrographs of peptide induced changes on bacterial membrane. Description of panels (A) untreated *S*. *aureus* cells (B) bleb (C) bud and (D) negative curvature formation on *S*. *aureus* membrane. (E) Untreated *S*. *typhimurium* cells (F) Negative curvature (G) bud (H) pore like structure formation on *S*. *typhimurium* surface. (I-L) TEM images showing (I) untreated cells and (J) bud formation on *S*. *aureus* membrane. (K) untreated *S*. *typhimurium* cells and (L) wrinkling and formation of pore like structure on *S*. *typhimurium* membranes.

Scanning and transmission electron microscopy of G+ (*S*. *aureus*) and G- (*S*. *typhimurium*) bacteria revealed three broad categories of anomalies in the morphology and membrane architecture: swelling of cells, formation of bleb/buds, and formation of concave curvature on bacterial membrane (Fig 5). At lower concentrations (≤2× MIC), buCATHL4B, 4C, 4F, and 4G caused swelling and blebing of *S*. *aureus* cells (Fig 5A). At increasing concentrations (>2× MIC), buCATHL4B, 4F, and 4G caused budding of both *S*. *aureus* and *S*. *typhimurium*. Formation of negative curvature, induced by buCATHL4B, 4C, 4D, and 4E, was more evident with *S*. *typhimurium*. Formation of pore like structures over the membrane was observed with buCATHL4B and 4C along with extensive wrinkling of membrane (Fig 5H and 5L). It has been shown that arginine rich peptides can lead to the formation of “negative Gaussian curvature” (or Saddle shaped curvature) on membrane through a robust interaction between guanidine group of arginine and multiple lipid headgroups [33–35]. The saddle shaped curvature is a topological requirement for the formation of toroidal pores and membrane protrusions, such as blebs and buds [36–38]. It was evident that buCATHL4s could induce such kind of changes on the bacterial membranes, which were dependent upon peptide concentration, incubation time, and type of bacteria (G+ /G-).

**Fig 6:**
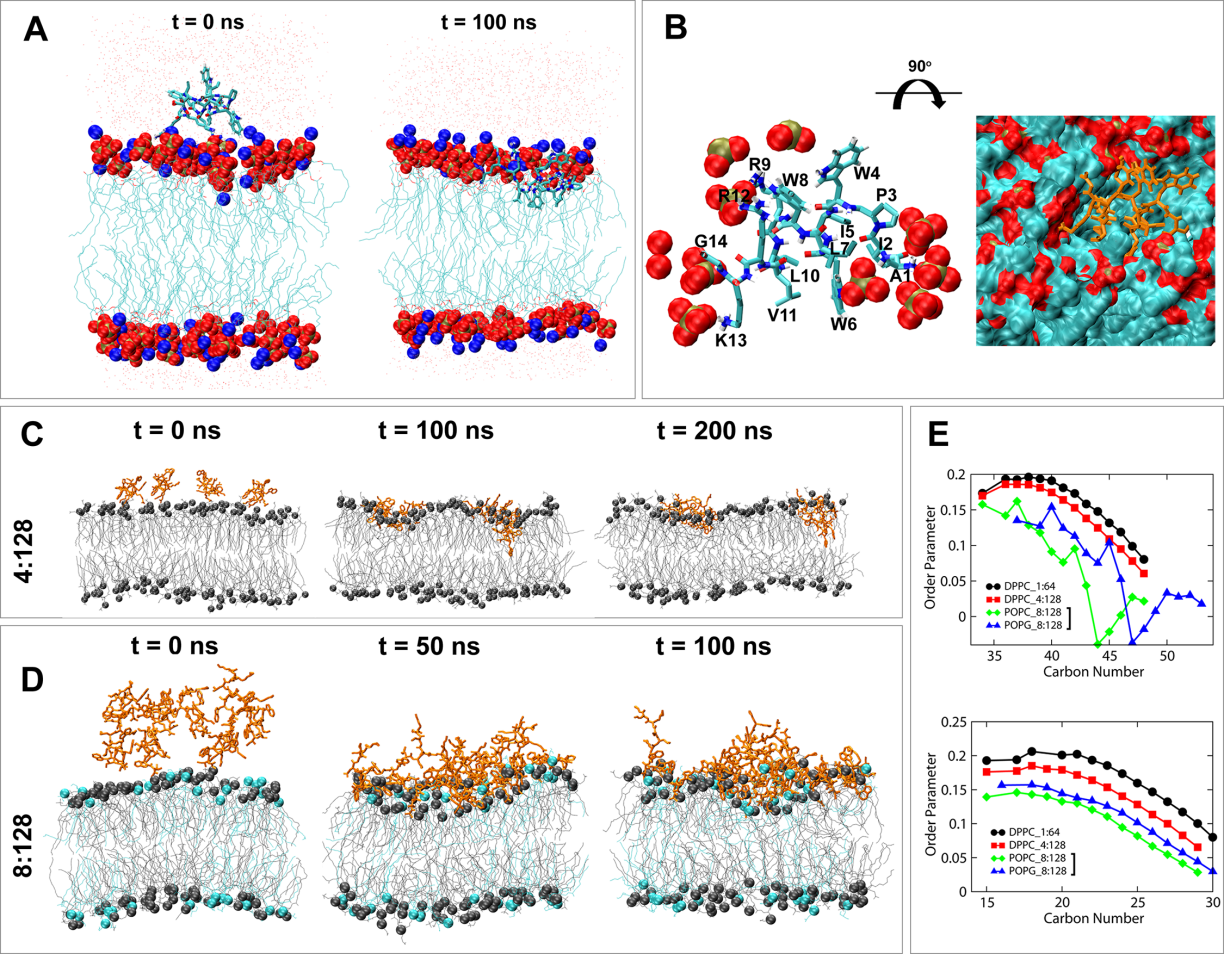
Binding of buCATH4B with model phospholipid membranes. (A) Single buCATH4B and 64 DPPC lipids at starting configuration (left) and at the end of 100 ns simulation (right). The peptide is modeled as stick; water molecules are shown as point; phosphate, oxygen, and nitrogen atoms are colored tan, red and blue, respectively. (B) Interaction of buCATH4B with phosphate groups present around 5 Å of the peptide (left), and orientation of buCATH4B (orange) on the DPPC membrane (right). (C) Four buCATH4Bs and 128 DPPC lipids, captured at starting configuration (left), at 100 ns (middle), and at the end of 200 ns simulation (right). Peptides are colored in orange and membrane in grey. Phosphate atoms are shown as grey spheres. (D) Eight buCATH4Bs and a 31 mixture of 96 POPC (grey) and 32 POPG (cyan) lipids. Peptides are colored in orange. Phosphate atoms of POPC and POPG are shown as grey and cyan spheres, respectively. (E) Order parameter (*Scd*) of the carbon atoms along the *sn-1* (upper) and *sn-2* (lower) acyl chain of phospholipids used in the simulation studies. Lower *Scd* value indicates more disordered lipid tails and *vice versa*.

To further explain the peptide-mediated disruption of bacterial membrane we studied the interaction of a representative peptide, buCATHL4B, with pre-equilibrated phospholipid membranes using molecular dynamics (MD) simulations (Fig 6). The Peptide:Lipid (P:L) ratios ranging from 1:64 to 8:128 were considered for simulation studies. At low concentration (P:L ratio 1:64), the peptide was quickly absorbed into the surface of the upper leaflet and located itself underneath the polar headgroups of the DPPC bilayer (Fig 6A). The electrostatic interactions between phosphate-oxygen and nitrogen atoms of arginine and lysine were crucial for initial absorption of the peptide on the membrane surface. The peptide displayed a nearly parallel orientation to the plane of the bilayer. At this orientation, the hydrophobic residues faced toward the hydrophobic interior while arginine and lysine formed hydrogen bonds with the neighboring phosphate groups (Fig 6B). At increasing concentrations (P:L ratios 2:64 and 4:128), the peptides formed dimer or higher order oligomers before being absorbed into the membrane with a similar orientation and position as was observed for the monomeric peptide (Fig 6C). This suggests that the peptides act cooperatively maintaining a consistent intermolecular interaction to ultimately execute membrane disruption. The same phenomenon was also observed with a negatively charged POPC:POPG membrane (Fig 6D). Spontaneous aggregation of peptides was observed in most of the simulations with the P:L ratios between 1:32 and 1:16, indicating the molecular aggregation is a regular phenomenon at higher concentration. The aggregation of large numbers of positively charged peptides at the upper leaflet of the membrane created a huge charge imbalance, which gradually pushed the headgroups of the upper leaflet downward and attracted the phosphate groups and glycerol moieties present at the lower leaflet of the bilayer. Understandably, this led to serious destabilization of the membrane, which was indicated by decreased thickness of the bilayer and increased disorderedness of lipid acyl chains (Fig 6E). At increasing concentrations aggregated peptides created local depressions on the membrane surface allowing water molecules trespass the lipophilic barrier from both leaflets of the bilayer (S1 Movie).

### buCATHL4s Assume Different Conformations in Aqueous and Membrane-Mimicking Environments

**Fig 7:**
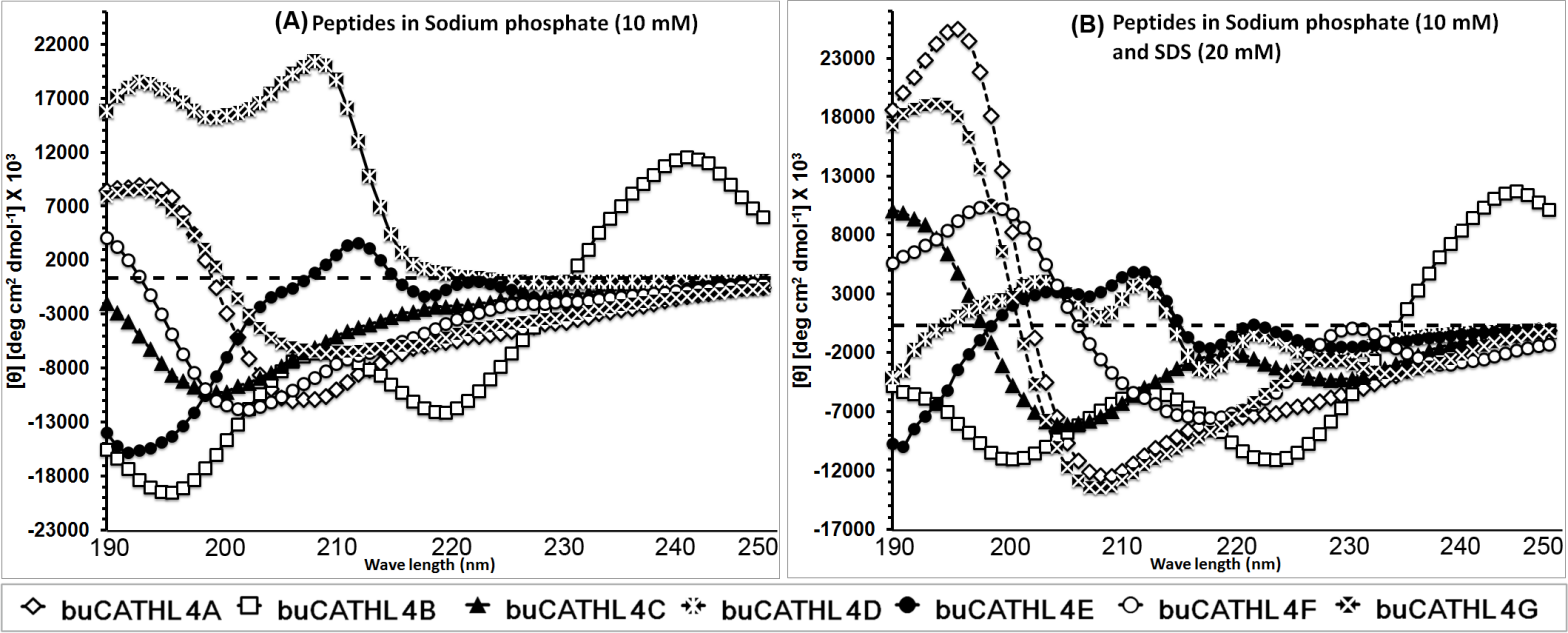
Circular dichroism (CD) spectroscopy of selected peptides in sodium phosphate (10 mM) with or without sodium dodecyl sulphate (SDS). buCATHL4s assumed backbone orientation ranging from random coil to alpha helix in water. The helical propensity increased further in presence of SDS micelles.

Natural cathelicidin peptides adopt flexible random structures as well as more rigid α-helical or β-sheet conformations. In aqueous buffer, buCATHL4C, 4E, and 4F showed a random backbone orientation with a flat base line up to 220 nm and a characteristic minimum at ~200 nm (Fig 7A). The buCATHL4D showed an extended sheet conformation with positive ellipticity signature. buCATHL4A, 4B, and 4G showed differential signatures by exhibiting a varied signal in the range of 235 to 205 nm, suggesting these peptides have a propensity to adopt a partially helical conformation in aqueous buffer (Fig 7A). It is interesting to note that the helical propensity for these peptides increases in the presence of helix forming amino acids as evidenced by the peptides 4F and 4G. A change of one amino acid Arg (4F) to His (4G) in the core of sequence increased the structural content of 4G following the Chou-Fasman analysis (Fig 7A). Indeed, the helical propensity of buCATHL4A and 4G increased further in presence of SDS micelles (Fig 7B). buCATHL4C and 4F exhibited a varied structural transitions in presence of SDS micelles, but such conformational shift was not observed for buCATHL4B. Peptides buCATHL4D and 4E exhibited unstructured conformation in presence of SDS.

The increase of the negative CD ellipticity of some of these peptides in the observed wavelength range can be directly attributed to one of the following phenomena or a combination of all: (i) An increase of the helical conformation of the peptides due to their interaction with SDS. In aqueous environment, SDS molecules form micellar rings around the transmembrane helical region of a membrane protein, mimicking the structure of the natural membrane bilayer [39]. (ii) The stacking interaction of the tryptophan’s aromatic (indole) rings with the peptide backbone [40]. It is worth noting that this phenomenon substantially interferes in calculation of percentage increase of helicity of these peptides by simple de-convolution methods applied for CD spectroscopy [40]. (iii) As the peptides showed a tendency to aggregate in biological assays, the signal modulations observed in aqueous and SDS micelles can be a result of differential aggregation behaviours in the observed buffers. In fact, a combination of several of these properties has been noticed in the MD simulations performed on buCATHL4B.

### buCATHL4s Peptides Are Cytotoxic at Higher Concentration, but Induce an *In-Vitro* Pro-Inflammatory Cytokine Response at Lower Concentration

**Fig 8:**
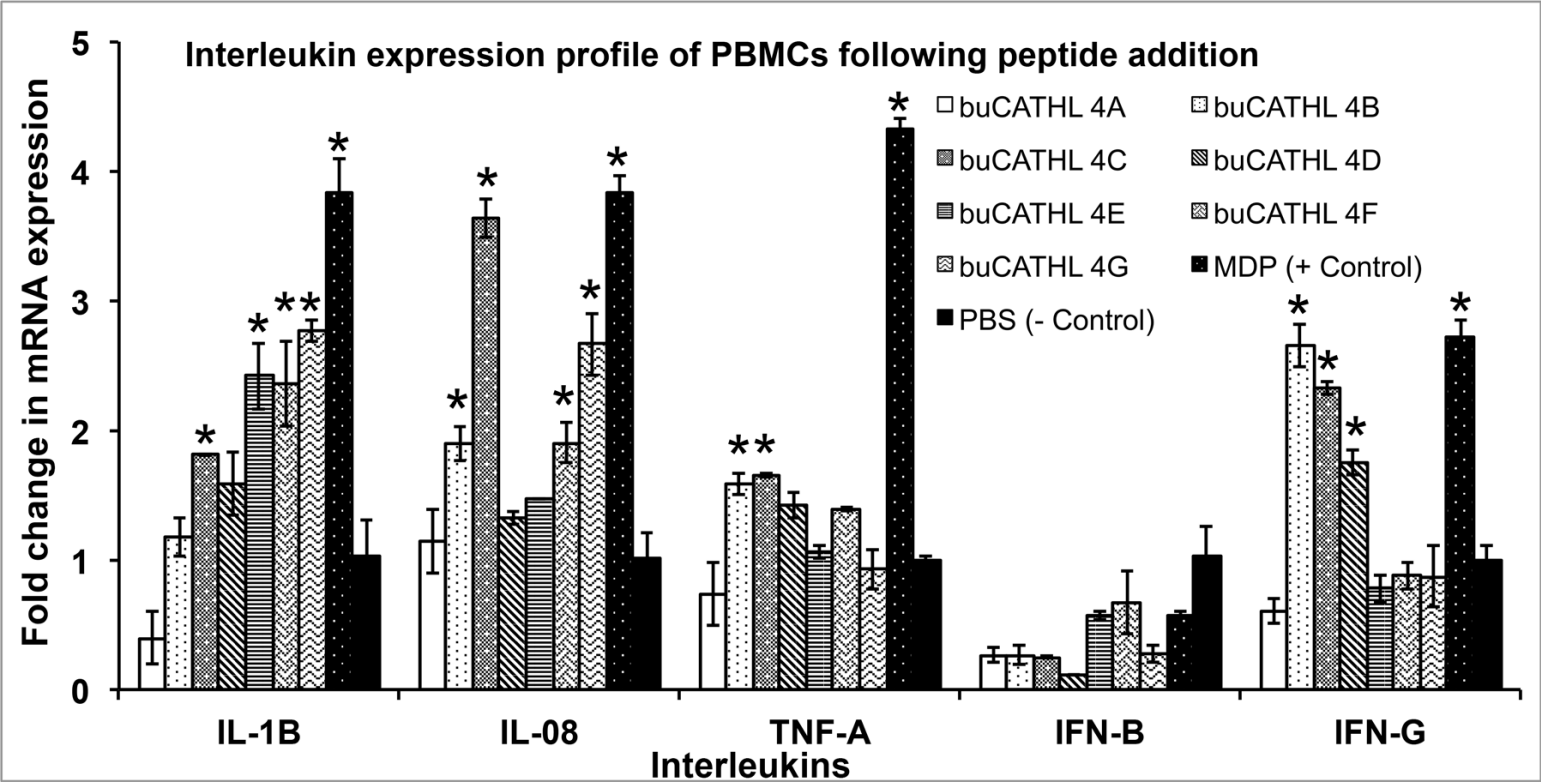
Relative fold change in mRNA expressions of cytokines following addition of peptides. Muramyl di peptide (MDP) and PBS were positive and negative controls, respectively. Columns with asterisk (*) had significantly (p<0.05) higher expression level over negative control.

Apart from their direct microbicidal action, antimicrobial peptides are known to mediate host immune response [41]. To investigate immunomodulatory properties of buCATHL4s, cytokines profile of cultured PBMCs was measured after peptide addition (500 nM). We found elevated mRNA levels of pro-inflammatory cytokines like IL-1β, IL-08 and TNF-α although no significant rise in IFN-β level was observed (Fig 8). Comparatively, amplitudes of IL-1β, IL-08 responses were more evident for all the peptides than TNF-α response, which was significantly higher for buCATHL4B and 4C only. No detectable level of anti-inflammatory cytokine IL-10 was observed either in control or treated cells. A notable observation was that the peptide induced IFN-γ response in cultured lymphocytes. This can be attributed to a fairly rich proportion of γδ+ T cells in bovine lymphocyte population [42]. γδ+ T cells are innate source of IFN-γ and can induce IFN-γ response without professional antigen presenting cells (APCs) [43]. Together, these evidences are suggestive of the pleiotropic action of buCATHL4s besides their ability to kill pathogens.

**Fig 9:**
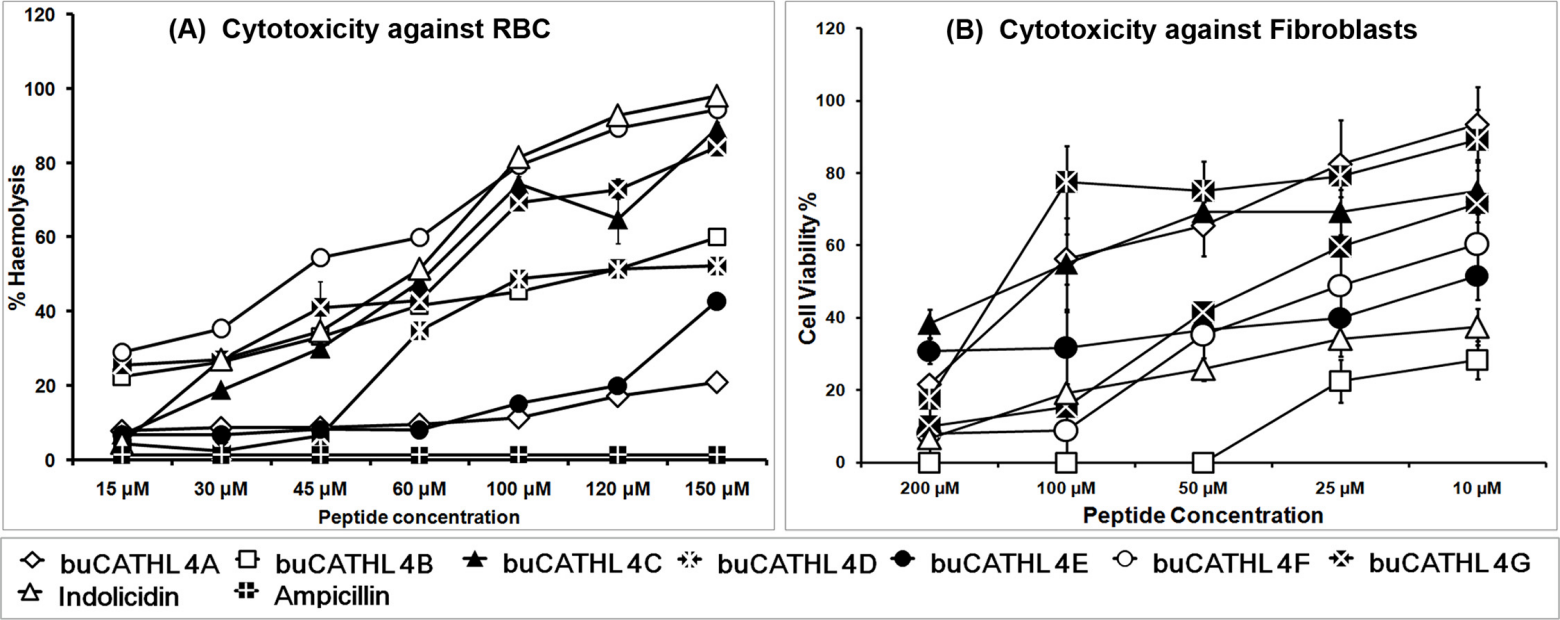
Cytotoxicity profiles of CATHL4 AMPs against RBC and fibroblast cells. (A) Haemolysis percentage of buffalo red blood cells following addition peptides at different molar concentrations. (B) Percentage of propidium iodide (PI) positive fibroblast cells following addition of peptides at different molar concentrations.

To assess untoward effects of peptides on eukaryotic cells, we measured cytotoxicity of buffalo CATHL4 peptides towards RBC and skin derived foetal fibroblast cells. buCATHL4A, 4D, and 4E showed relatively low toxicity towards RBC showing less than 10% haemolysis at 45μM concentration (Fig 9A). Comparatively, the buCATHL4A and 4E were less toxic even at high concentration (120 μM). At concentrations above 45μM, buCATHL4B, 4G, 4H caused rapid haemolysis. However, it has to be noted that haemolytic concentration of peptides were well above the MIC. As with RBC, buCATHL4A showed less toxicity towards fibroblast cells, even at higher (200μM) concentration (Fig 9B; Fig G in S1 File). Proportion of PI positive cells were comparatively lower (20–28%) with peptides buCATHL4C, 4D and 4E at this concentration (200μM). However, buCATHL4B showed a higher proportion of PI positive cells even at 10μM concentration and at concentrations above 50μM, membrane permeability of nearly whole cell population were affected. Similar observation was also found with buCATHL4G and 4H, showing a high proportion of PI positive cells at concentration above 25μM. Proportion of fibroblast cells showing complete lysis was very negligible even at high concentration of peptides. Briefly, selective buCATHL4 peptides could be considered less toxic to fibroblast cells and were reasonably safe to RBC at therapeutic concentrations.

## Discussion

The ecological niche of buffalo with high microbial burden in their natural habitat necessitates a strong counter mechanism for survival. Therefore, it is not surprising that cathelicidin, a component of primary defence, has undergone diversification in buffalo as seen in other species (*e*.*g*. pig, monotremes etc.). It is intriguing why this particular group has been selected naturally for diversification. A possible explanation could be that a pathogen-driven selection for resistance played a role, endowing superior antimicrobial spectrum and potency to the newly identified buCATHL4s over the prevalent wild type (buCATHL4A). The broad-spectrum antimicrobial action of new buCATHL4 variants against both G+ and G- pathogens indicate that they were naturally selected as a component of primary defence in buffalo. In particular, G- bacteria are often insensitive to drugs because of their perfect three-tier barriers. The outer membrane is a barrier for amphipathic compounds [44]; the multidrug-resistant pumps extrude compounds that leak in through the outer membrane [45]; and the inner membrane restricts penetration of hydrophilic substances [46]. Additionally, bacteria can develop resistance against drugs by acquisition of resistance genes through horizontal transfer [47]. It is interesting to notice that buCATHL4s have maintained their antimicrobial properties over millions of years while bacteria could quickly develop resistance against antibiotics [48]. This could have been possible because the bacterial killing mechanism of buCATHL4s is associated with disruption of the bacterial cell membrane, which is an immutable component. There exist hypotheses regarding bactericidal mechanism of AMPs [49], but establishment of structure-activity relationships between AMPs and their microbicidal action have remained elusive. This study shows that at least part of bacterial killing mechanisms by buCATHL4s is mediated through destabilization and disruption of the cell membrane leading to formation of negative curvature, bleb, bud, and pore-like structures. It will be intriguing to know whether the peptides have other intracellular targets, as documented for cattle indolicidin that binds to the intracellular DNA and protein for its antimicrobial action [6, 50].

It was evident that a few changes in amino acids in the buCATHL4s contributed to immense variation in biological and structural properties of the peptides. The higher MIC values observed for native buCATHL4A peptide could be attributed to presence of fewer hydrophobic and charged residues, which are necessary for amphipathic and membrane penetrating properties. Other buCATHL4 variants showed presence of more tryptophan and arginine residues in their AMD and can be structurally classified into two classes. The first class of peptides (class I; buCATHL4B, 4E, 4F and 4G) has a characteristic hydrophobic N-terminal portion and the positively charged C-terminal flanking end. As evidenced in the simulation study, in a lipid environment these peptides can orient themselves by exposing their charged residues towards the phosphate groups in order to capture the electrostatic interactions and further self-associate/oligomerize using their hydrophobic portions. Such an oligomerized peptide in the presence of lipid environment can be envisaged as a “brush” where the hydrophobic region serves as a shaft and the charged part serves as a flanking surface. The second class of peptides (class II; buCATHL4C and 4D) possesses the above mentioned properties of class-I peptides, but is further equipped with positively charged residues preceding the hydrophobic portion at the N-terminus. Such a charged N-terminal flanking extension results in a “double-sided brush”. The inherent advantage of such a flanking charge on both sides is higher adaptability against different types of lipid/membrane environments.

For both classes of peptides, the orientation with respect to the membrane is facilitated by the basic guanidine groups of arginine, responsible for strong hydrogen bonding and electrostatic interactions with the polar headgroup of phospholipids. Further, the bulky aromatic and partially polar side chain of tryptophan is essential for insertion of peptide into lipophilic region of membrane [51]. Indeed, our MD simulation studies showed that the arginine and the tryptophan residues together assist in initial absorption and translocation of buCATH4B within the membrane surface in a seemingly energy independent manner. The guanidine group of arginine was pivotal for initial anchoring with the phospholipid headgroups. This makes the randomly placed peptides to obtain an initial amphipathic orientation where the tryptophan side chains gradually moved deep into the hydrophobic core of the bilayer to lock a desired low energy conformation. Thus, it is apparent that natural selection of arginine and tryptophan has given new buCATHL4 variants more amphipathicity and membrane penetrating properties.

The properties of buCATHL4 peptides such as shorter length, potent antimicrobial action, and low cytotoxicity also emphasize their clinical use for development of novel antibiotics and or peptidomimetics. Among new variants, buCATHL4F and 4G showed good potency towards G+ pathogens and *S*. *typhimurium*. The peptides were relatively quick in eliminating *S*. *aureus* and *P*. *aeruginosa*, and induced an *in-vitro* proinflammatory cytokine response in PBMCs. However, the peptides showed no marked effect against growth of biofilms. Another potential limitation of buCATHL4F and 4G was higher MICs against G- organisms (except *S*. *typhimurium*), where cytotoxicity level of the peptides were also very high. The rest four peptides (buCATHL4B, 4C, 4D, and 4E) were very potent against both G+ and G- pathogens and showed activity against stationary phase culture. Barring buCATHL4B, cytotoxicity of the peptides at therapeutic concentration was negligible against both RBC and fibroblast cells. However, low efficiency against reduction of bacterial biofilm growth by buCATHL4B and 4E could limit their therapeutic use against chronic infections. Fortunately, the above limitations were not associated with buCATHL4C and 4D. Bacterial clearance time for buCATHL4C, in particular, was rapid and comparable or better than ampicillin or indolicidin. The effect of these two peptides against biofilm growth was remarkable. Additionally, both the peptides were capable of initiating a pro-inflammatory cytokine response. It is noteworthy that the amino acid sequences of buCATHL4C and 4D are essentially the same except two substitutions at their C-terminal part. The substitution of valine (buCATHL4C) to phenylalanine (buCATHL4D) at position 16 can be considered insignificant owing to similar and inherent hydrophobic characteristics of these amino acids. The other modification (Arginine in buCATHL4C to Proline in buCATHL4D) at position 14 could significantly modulate the biological and structural properties of the peptide as arginine has comparatively stronger interaction properties with the membrane lipid phosphate groups, and loss of arginine can contribute to the differential binding phenomenon with membrane [33]. The structural behaviour of these two peptides obtained from CD spectroscopy was essentially different and could be related to the presence of proline at position 14 in 4D, which remained in the extended conformation even in the presence of SDS micelles. Moreover, our experiments demonstrated that the antimicrobial potency and bacterial clearance time for buCATHL4C were better compared to 4D and also to class-I peptides. Here, we envisage that the “RR” motif on the C-terminal basic patch of 4C along with its two-sided brush-like arrangement of positive charges is responsible for its enhanced performance as an antimicrobial agent. Further, considering similar sequence and structural features observed for sequences in this current study, we presume that positional dependence of the “positively charged residues” along the sequence also significantly contributed to the differential antimicrobial, agglutination and cytotoxic properties. However, a detailed future study deciphering the structural and sequence significance of these charged residues is essential to gain more insights into their differential activities. In summary, cathelicidin genes and CATHL4, in particular, have undergone diversification in buffalo. AMPs of new cathelicidins variants possess robust antimicrobial activity and exert their action by disrupting bacterial membrane integrity. Ability to induce a pro-inflammatory response suggests a pleiotropic role of the peptides in innate immunity of buffalo. Further, suitability of in-house synthesis (short peptides devoid of disulfide bridges) and low cytotoxicity towards eukaryotic cells at bactericidal concentration make them potent candidates for development of novel topical antibiotics and antimicrobial peptidomimetics.

## Supporting Information

S1 File Text A: Method: MD Simulations. **Table A:** Primers used for amplification of full length buffalo cathelicidin genes. **Fig A**: Repertoire of cathelicidin family in mammalian species. Human has only one cathelicidin gene (CAMP) that encodes LL-37 peptide. In contrast, horse, pig and ruminants harbor multiple copies of the genes that may encode similar or different types of mature peptide. Thus, pig has four main cathelicidin types but total eight cathelicidin genes with two copies of PMAP36 and four copies of PG/NPG genes. Cattle and buffalo have seven cathelicidin types, but our study suggests expansion of *CATHL4* has occurred in buffalo, thus total number of cathelicidin genes is likely to be higher in buffalo. The diagram is based on information available at UCSC genome browser. **Fig B**: Different Single Strand Conformation Polymorphism (SSCP) patterns of *CATHL4*exonIV from clones of multiple animals. **Fig C**: Comparison of PCR-SSCP band patterns in allelic versus copy number variation. As per previous and our observations, chances of bacterial clone carrying two or more types of plasmids are extremely rare due to isolation of nuclear haplotypes during cloning and SSCP (Scharf et al., 1986; Orti et al., 1997). Therefore, generally two and maximum three SSCP patterns could be found for an animal carrying heterozygous alleles even at more than one locus (two loci shown in the figure). However, if these loci are on different copies of a gene, at least four band patterns will be observed, even the animal is homozygous for all the alleles. **Fig D**: SSCP pattern of *CATHL4*exonIV from multiple clones of a single animal. Maximum three (homozygous dominant, homozygous recessive and heterozygous) patterns should be observed for an allele. More than three patterns for *CATHL4* gene were observed here, indicating possible duplication of the gene. A SSCP pattern was only considered when it was present in at least two lanes. Lanes marked with asterisk (*) were either present as singlet or missed double stranded DNA and were not included in analysis. **Fig E**: Standard curves and amplification curves of absolute quantitation by qRT-PCR. CATHL4 showed a marked variation in Cp values for different breeds of buffalo. A parallel experiment with buffalo CATHL5 revealed no significant variation in the Cp values for different breeds. **Fig F**: Changes in membrane permeability of stationary phase of *S*. *aureus*, *P*. *aeruginosa* and *S*. *typhimurium* cultures following peptide addition. Green and red fluorescence indicates live and dead cells, respectively. **Fig G**: Peptide induced changes in the membrane permeability of buffalo foetal fibroblast cell culture. Channel P2 indicates proportion of PI negative normal cells with intact plasma membrane, whereas channel P3 indicates proportion of PI positive cells with altered membrane permeability.
(PDF)

S1 Movie Representative movie clip showing trespassing of water through the membrane during 96–100 ns MD simulation. Peptides are modeled orange stick, lipids are grey lines, phosphorous atoms are grey spheres, and water molecules are red spheres.
(MPG)

## Abbreviations

AMD: Antimicrobial domain
AMPs: Antimicrobial peptides
CFU: Colony forming unit
DPPC: Dipalmitoyl-sn-glycero-3-phosphocholine
gDNA: Genomic DNA
MIC: Minimum inhibitory concentrations
OD: Optical density
PBMCs: Peripheral blood mononuclear cells
POPC: 1-palmitoyl-2-oleoylphosphatidylcholine
POPG: 1-palmitoyl-2-oleoyl-sn-glycero-3-phosphoglycerol
qRT-PCR: Real time polymerase chain reaction
SNV: Single nucleotide variation
SSCP: Single strand conformational polymorphism
