# Supplementary material for: Diversity, Antimicrobial Action and Structure-Activity Relationship of Buffalo Cathelicidins
Source: PLoS One. 2015 Dec 16;10(12):e0144741. doi: 10.1371/journal.pone.0144741 (PMC4684500; doi:10.1371/journal.pone.0144741)

Supplementary Information

Diversity, antimicrobial action and structure-activity relationship of buffalo cathelicidins

Biswajit Brahma, Mahesh Chandra Patra, Satyanagalakshmi Karri, Meenu Chopra, Purusottam Mishra, Bidhan Chandra De, Sushil Kumar, Sourav Mahanty, Kiran Thakur, Krishna Mohan Poluri, Tirtha Kumar Datta and Sachinandan De.

Animal Genomics Lab, Animal Biotechnology Center, National Dairy Research Institute, Karnal 132001, Haryana, India.

Supplementary Methods:

Text A:

Method: MD Simulations

MD simulations were performed to study the mechanism of membrane deformation in the order of 100 ns time scales. For this study, buCATH4B which showed the lowest MIC was modeled using Discovery Studio 3.5 freeware (Accerlys software Inc.) based on the secondary structure obtained from CD. Different compositions of the modeled buCATH4B were simulated with model phospholipid membranes consisting of either the zwitterionic 1,2-dipalmitoyl-sn-glycero-3-phosphocholine (DPPC) or a more negatively charged 3:1 mixture of 1-palmitoyl-2-oleoylphosphatidylcholine (POPC) and 1-palmitoyl-2-oleoyl-sn-glycero-3-phosphoglycerol (POPG) lipids. The membranes were equilibrated for 40 ns before adding peptides. The corresponding Protein:Lipid (P:L) ratios of the simulation systems were 1:64, 2:64, 4:128, and 8:128. Multiple simulations were performed for each system with different initial random velocities assignments. The peptides were initially placed asymmetrically in the aqueous phase close to one of the leaflets of the bilayer.

All MD simulations were performed using the GROMACS 4.5 software package. The Berger-

lipid force field was used for phospholipids and Gromis96-53a6 parameters were used to represent the peptide interactions. The short range (Lennard-Jones) interactions were calculated using a twin range cutoff distances of 1.0/1.4 nm and a pair list update frequency of once per 10 steps. Long range (electrostatic) interactions were calculated using particle mesh Ewald (PME) method. PME is very common and considered the most efficient method in computing electrostatic interactions. On the other hand, there have been reports that truncating electrostatic interactions (using cut-off distances) led to serious distortions in the bilayer properties, such as, artificial ordering of polar headgroups of DPPC molecules [1]. The SPC water molecules were used to solvate the simulation systems. The time step for all simulations was 1 fs. The LINCS algorithm was used to constrain the bond lengths. The simulations were performed in an NPT ensemble with the application of periodic boundary condition. The temperature was coupled (coupling time 0.2 ps) through Nose-Hoover thermostat to 323 K for DPPC, 271 K for POPC, and 269 K for POPG lipids, to be well above the main-phase transition temperatures of the respective phospholipids. The pressure was coupled using semi-isotropic coupling scheme with a coupling time of 0.5 ps and compressibility of 5 x 10-5 bar-1. Trajectory data analysis and structure visualization were performed using VMD and accessory plugins.

Table A: Primers used for amplification of full length buffalo cathelicidin genes

Gene	Primer name	Sequence (5′ – 3′)	Length (bp)	T_m (^0C)
CATHL1	CATHL1FW	TCAGACTGGGCACCATGGAGACC	23	61
	CATHL1RV	CGCGCATGTCACAAGAATTTATTTTTCAGATCC	33	61
CATHL2	CATHL2FW	GGAGACTGGGGACCATGGAGACC	23	62
	CATHL2RV	TCCCAAGAGGTCTTCCCTGGGCT	23	61
CATHL3	CATHL3FW	GGAGACTGGGGACCATGGAGACC	23	62
	CATHL3RV	GCTTCACAATGGCCTTGGAATTGGCC	26	61
CATHL4	CATHL4FW	GGAGACTGGGGACCATGGAGACC	23	62
	CATHL4RV	GTCACTGTCCAGAAGCCCGAATCTG	25	61
CATHL5	CATHL5FW	GGAGACTGGGGACCATGGAGACC	23	62
	CATHL5RV	CAGAAGTCTTCCCCAGGGCCGG	22	62
CATHL6	CATHL6FW	GGAGACTGGGGACCATGGAGACC	23	62
	CATHL6RV	TCCATGGGCTCACAATTTACCCCAAATG	28	60
CATHL7	CATHL7FW	GGAGACTGGGGACCATGGAGACC	23	62
	CATHL7RV	GTCAGAGTTCAGAAATCTGAGCCAGGATAAC	31	62

Primers used for SSCP analysis of *CATHL4*.

Gene	Primer	Sequence (5′ – 3′)	Length (bp)	T_m (^0C)
CATH4	Forward	GCTTCCTTTTTCACAGCTCCAGAGTGTC	28	61
	Reverse	GTCACTGTCCAGAAGCCCGAATCTG	25	61

Primers used for estimation of copy number variations.

Gene	Primer	Sequence (5′ – 3′)	Length (bp)	T_m (^0C)
CATH4	Forward	CTGTCAGATCCTGAGCCTGGGGAA	24	61
	Reverse	GTCACTGTCCAGAAGCCCGAATCTG	25	61
CATH5	Forward	GGGGACTTCGAAGCCTGGGTAGGA	24	63
	Reverse	CGGCCCACAATTCACCCAATTCTGATT	27	60

Supplementary Results:

Figure A: Genomic organization of cathelicidin genes in mammals. Human has only one cathelicidin gene (CAMP) that encodes LL-37 peptide. In contrast, pig and ruminants harbor multiple copies of the genes that may encode similar or different types of mature peptide. The diagram is based on information available at UCSC genome browser.

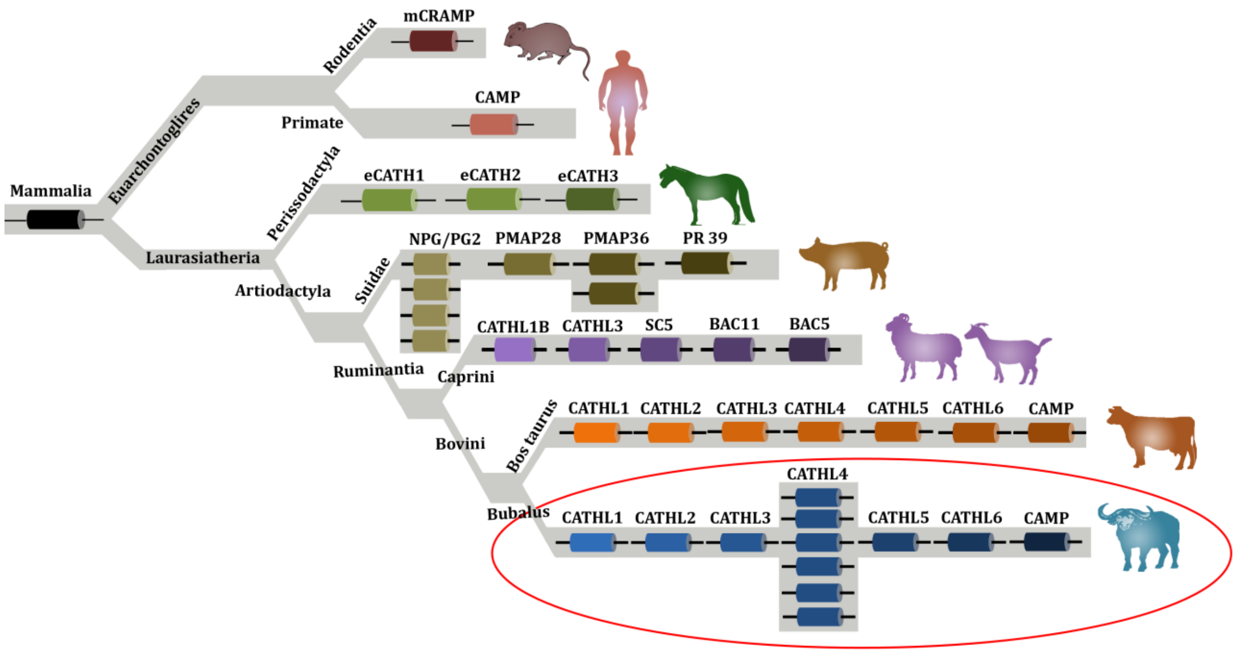

Figure B: Different Single Strand Conformation Polymorphism (SSCP) patterns of *CATHL4*exonIV from clones of multiple animals. The dsDNA at the bottom indicated two major subtypes *i.e.* shorter (lanes 1-11) and longer (lanes 12-17 and 19-22) *CATHL4* variants that differed by 12 bp.

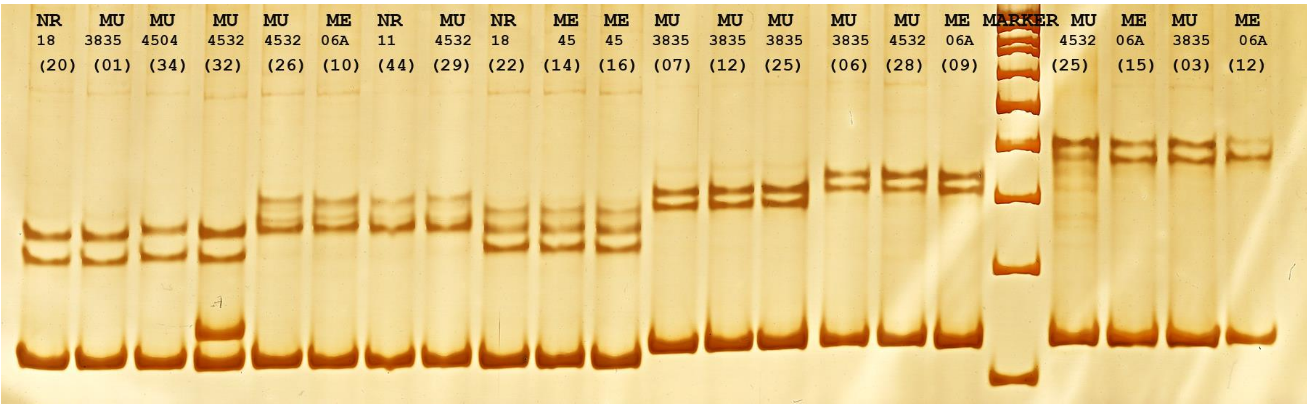

Figure C: Comparison of PCR-SSCP band patterns in allelic versus copy number variation. As per previous and our observations, chances of bacterial clone carrying two or more types of plasmids are extremely rare due to isolation of nuclear haplotypes during cloning and SSCP (Scharf et al., 1986; Orti et al., 1997). Therefore, generally two and maximum three SSCP patterns could be found for an animal carrying heterozygous alleles even at more than one locus (two loci shown in the figure). However, if these loci are on different copies of a gene, at least four band patterns will be observed, even the animal is homozygous for all the alleles.

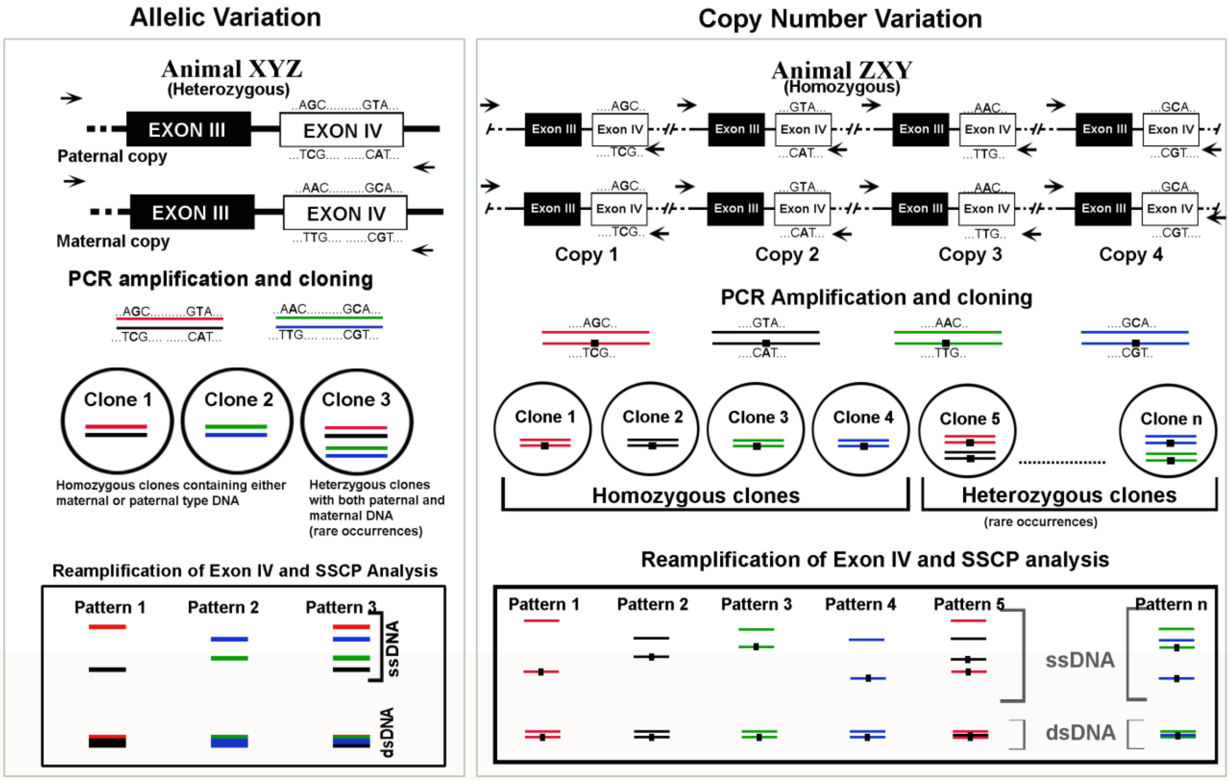

Figure D: SSCP pattern of *CATHL4*exonIV from multiple clones of a single animal. More than three SSCP patterns of *CATHL4* for a single animal suggested possible duplication of the gene. A SSCP pattern was only considered when it was present in at least two lanes. Lanes marked with asterisk (*) were either present as singlet or missed dsDNA were considered as PCR and cloning artifacts and were not included in analysis.

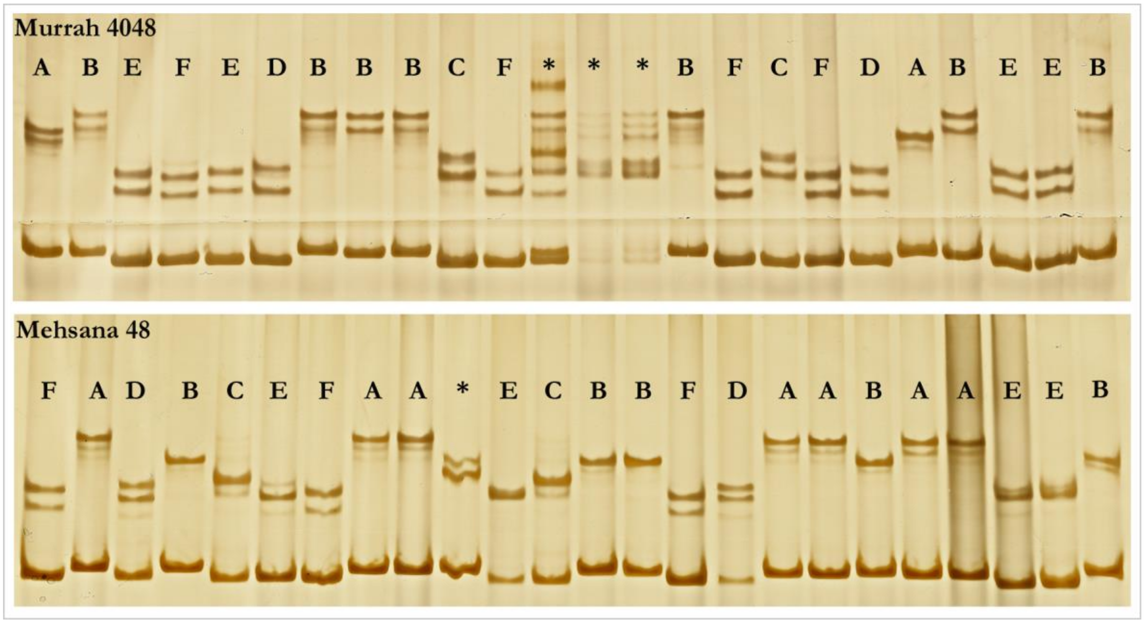

Figure E: Standard curves and amplification curves of absolute quantitation by qRT-PCR. CATHL4 showed a marked variation in Cp values for different breeds of buffalo. A parallel experiment with buffalo CATHL5 revealed no significant variation in the Cp values for different breeds.

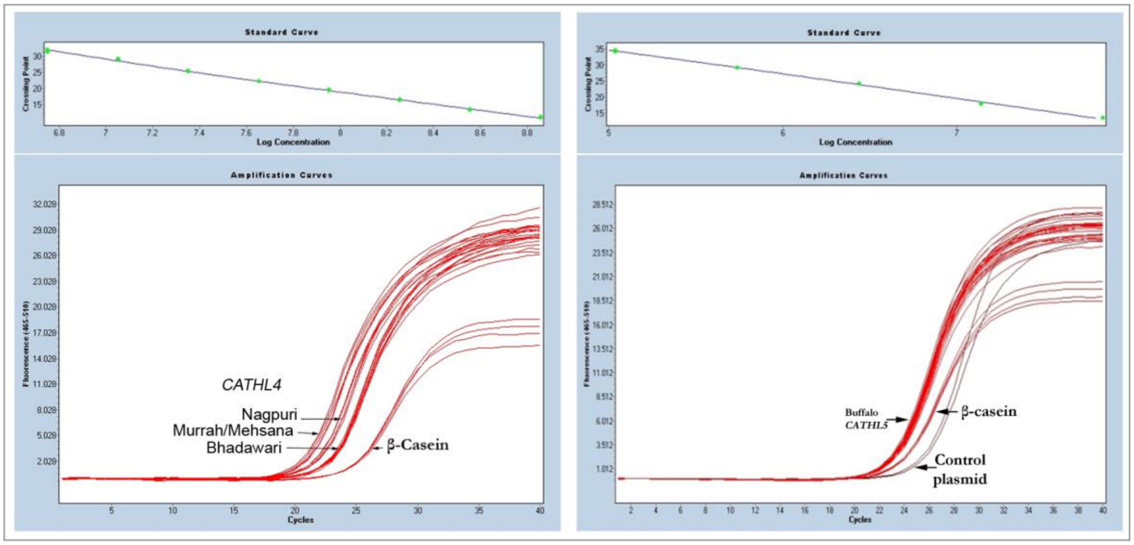

Figure F: Changes in membrane permeability of stationary phase of *S. aureus*,*P. aeruginosa* and *S. typhimurium* cultures following peptide addition. Green and red fluorescence indicates live and dead cells, respectively.

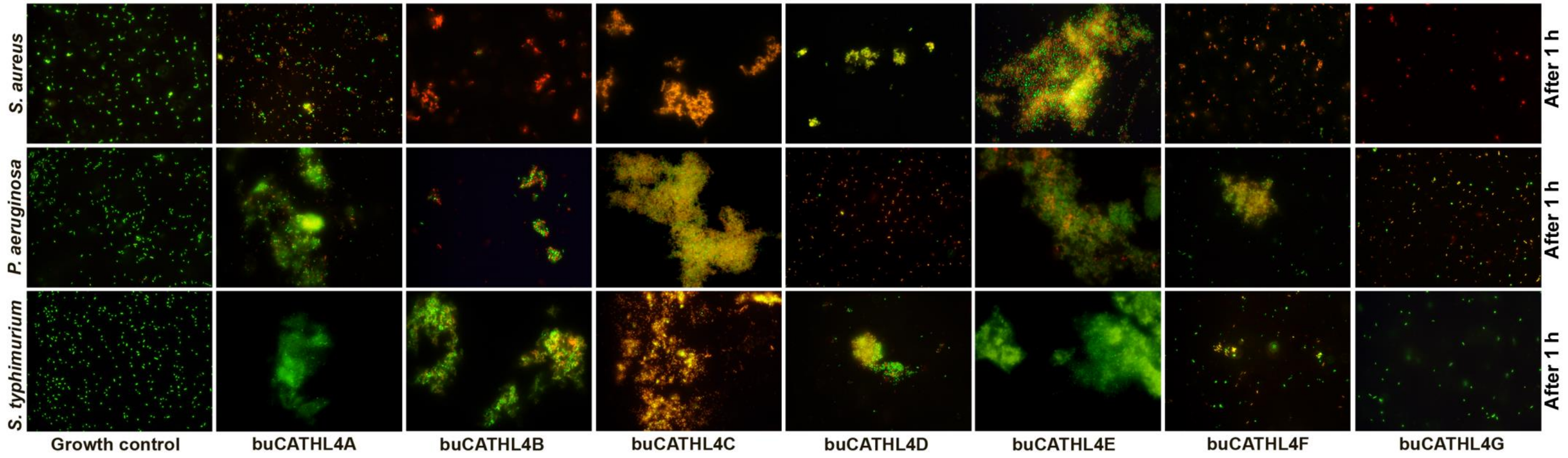

Figure G: Peptide induced changes in the membrane permeability of buffalo foetal fibroblast cell culture. Channel P2 indicates proportion of PI negative normal cells with intact plasma membrane, whereas channel P3 indicates proportion of PI positive cells with altered membrane permeability.

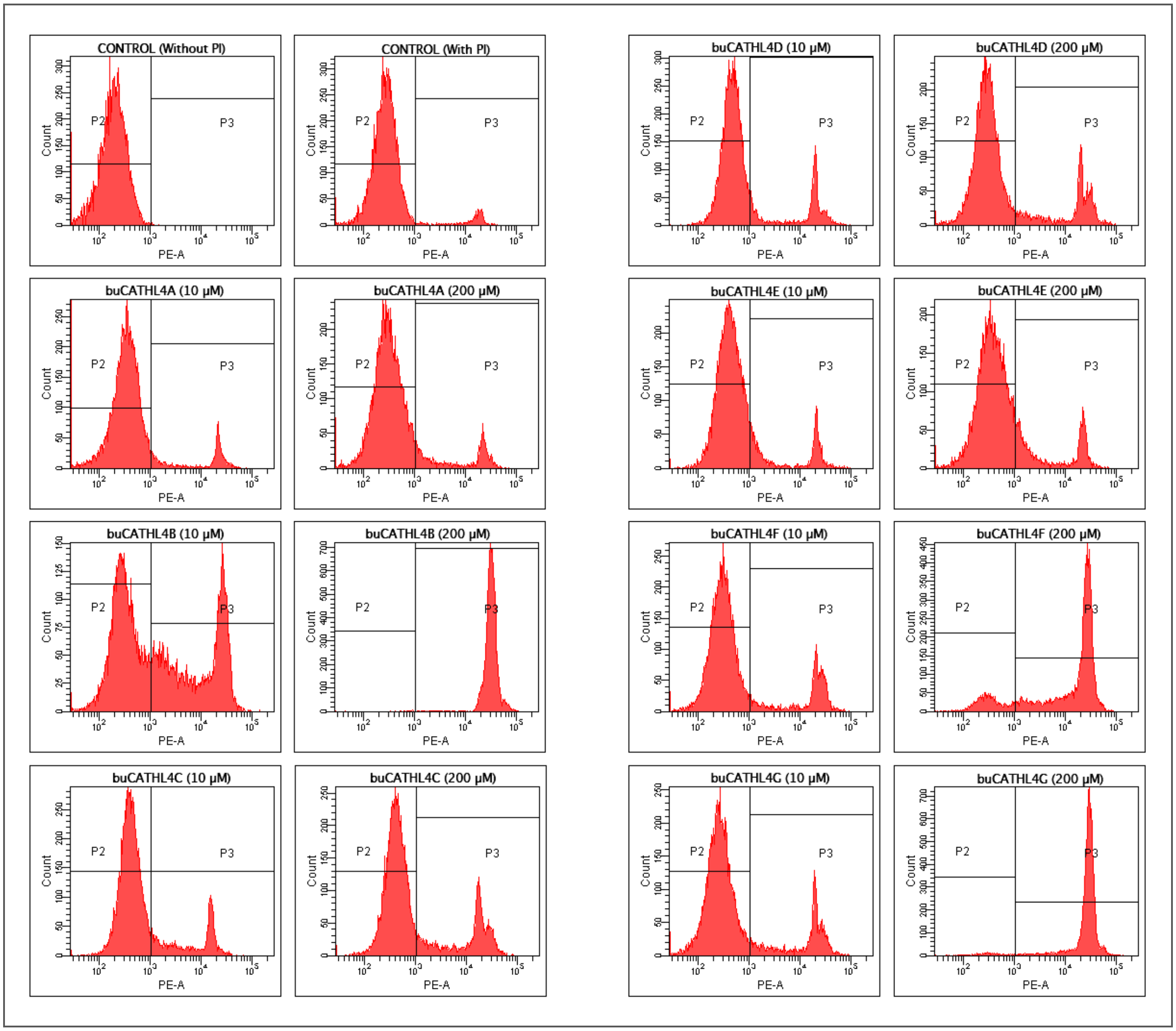

Movie caption:

Movie S1: Representative movie clip showing trespassing of water through the membrane during 96-100 ns MD simulation. Peptides are modeled orange stick, lipids are grey lines, phosphorous atoms are grey spheres, and water molecules are red spheres.

Still image from movie:

Still image frommovie showing trespassing of water through the membrane during MD simulation. Peptides are modeled orange stick, lipids are grey lines, phosphorous atoms are grey spheres, and water molecules are red spheres.

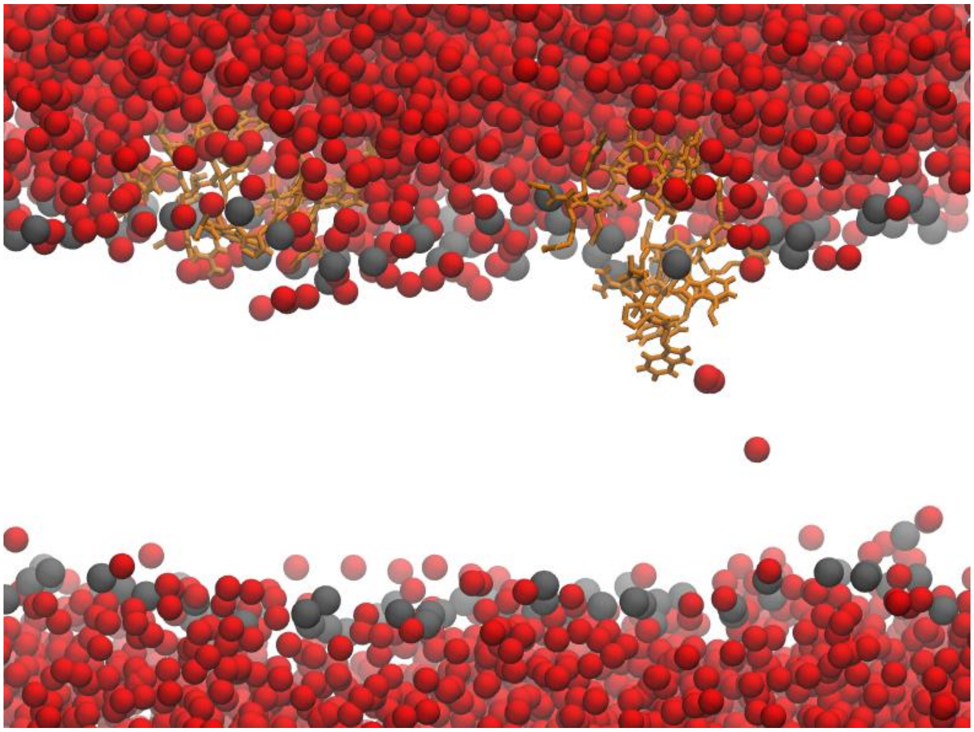

Supplement: S1 File — Table A: Primers used for amplification of full length buffalo cathelicidin genes. Fig A: Repertoire of cathelicidin family in mammalian species. Human has only one cathelicidin gene (CAMP) that encodes LL-37 peptide. In contrast, horse, pig and ruminants harbor multiple copies of the genes that may encode similar or different types of mature peptide. Thus, pig has four main cathelicidin types but total eight cathelicidin genes with two copies of PMAP36 and four copies of PG/NPG genes. Cattle and buffalo have seven cathelicidin types, but our study suggests expansion of CATHL4 has occurred in buffalo, thus total number of cathelicidin genes is likely to be higher in buffalo. The diagram is based on information available at UCSC genome browser. Fig B: Different Single Strand Conformation Polymorphism (SSCP) patterns of CATHL4exonIV from clones of multiple animals. Fig C: Comparison of PCR-SSCP band patterns in allelic versus copy number variation. As per previous and our observations, chances of bacterial clone carrying two or more types of plasmids are extremely rare due to isolation of nuclear haplotypes during cloning and SSCP (Scharf et al., 1986; Orti et al., 1997). Therefore, generally two and maximum three SSCP patterns could be found for an animal carrying heterozygous alleles even at more than one locus (two loci shown in the figure). However, if these loci are on different copies of a gene, at least four band patterns will be observed, even the animal is homozygous for all the alleles. Fig D: SSCP pattern of CATHL4exonIV from multiple clones of a single animal. Maximum three (homozygous dominant, homozygous recessive and heterozygous) patterns should be observed for an allele. More than three patterns for CATHL4 gene were observed here, indicating possible duplication of the gene. A SSCP pattern was only considered when it was present in at least two lanes. Lanes marked with asterisk (*) were either present as singlet or missed double stranded DNA and were [file pone.0144741.s001.pdf]
